# The effector repertoire of *Fusarium oxysporum* determines the tomato xylem proteome composition following infection

**DOI:** 10.3389/fpls.2015.00967

**Published:** 2015-11-04

**Authors:** Fleur Gawehns, Lisong Ma, Oskar Bruning, Petra M. Houterman, Sjef Boeren, Ben J. C. Cornelissen, Martijn Rep, Frank L. W. Takken

**Affiliations:** ^1^Molecular Plant Pathology, Faculty of Science, Swammerdam Institute for Life Sciences, University of AmsterdamAmsterdam, Netherlands; ^2^RNA Biology and Applied Bioinformatics Research Group and MAD: Dutch Genomics Service and Support Provider, Faculty of Science, Swammerdam Institute for Life Sciences, University of AmsterdamAmsterdam, Netherlands; ^3^Laboratory of Biochemistry, Wageningen UniversityWageningen, Netherlands

**Keywords:** label free proteomics, effectors, pathogenicity, virulence, xylem sap

## Abstract

Plant pathogens secrete small proteins, of which some are effectors that promote infection. During colonization of the tomato xylem vessels the fungus *Fusarium oxysporum* f.sp. *lycopersici* (Fol) secretes small proteins that are referred to as SIX (Secreted In Xylem) proteins. Of these, Six1 (Avr3), Six3 (Avr2), Six5, and Six6 are required for full virulence, denoting them as effectors. To investigate their activities in the plant, the xylem sap proteome of plants inoculated with Fol wild-type or either *AVR2, AVR3, SIX2, SIX5*, or *SIX6* knockout strains was analyzed with nano-Liquid Chromatography-Mass Spectrometry (nLC-MSMS). Compared to mock-inoculated sap 12 additional plant proteins appeared while 45 proteins were no longer detectable in the xylem sap of Fol-infected plants. Of the 285 proteins found in both uninfected and infected plants the abundance of 258 proteins changed significantly following infection. The xylem sap proteome of plants infected with four Fol effector knockout strains differed significantly from plants infected with wild-type Fol, while that of the *SIX2*-knockout inoculated plants remained unchanged. Besides an altered abundance of a core set of 24 differentially accumulated proteins (DAPs), each of the four effector knockout strains affected specifically the abundance of a subset of DAPs. Hence, Fol effectors have both unique and shared effects on the composition of the tomato xylem sap proteome.

## Introduction

Pathogens such as bacteria, fungi, oomycetes, protozoa and nematodes continuously challenge the plant immune system. Although most attacks are unsuccessful, sometimes plants are infected and disease develops. Diseases in crops do not only result in yield losses, but can also affect food-safety and quality (Agrios, 2005). To facilitate infection and host colonization, pathogenic microorganisms deploy small, secreted proteins, often called effectors (Win et al., 2012). Some effectors suppress or evade plant basal immunity thereby conferring Effector Triggered Susceptibility (ETS), while others manipulate host factors required for a sustained compatibility (Jones and Dangl, 2006; Win et al., 2012). To unravel how pathogens manipulate the host one needs to study effector action.

Among the most devastating plant pathogens are fungi and they pose a widespread threat to our crops (Fisher et al., 2012). Of the soil-borne fungus *Fusarium oxysporum*, pathogenic and host-specific forms have evolved that cause wilt disease or foot, root or bulb rot on a variety of economical important crops like cotton, banana, melon and tomato (Tjamos and Beckman, 1989; Michielse and Rep, 2009). The tomato-infecting form, *F. oxysporum* f.sp. *lycopersici* (Fol) is the causal agent of Fusarium wilt disease. Fol invades the roots and subsequently colonizes the xylem vessels, thereby compromising water transport resulting in wilting of the plant (Michielse and Rep, 2009). So, during early stages of infection the interface and communication between pathogen and host is largely confined to the xylem sap (Michielse and Rep, 2009). Since the xylem sap can easily be collected this pathosystem is perfectly suited to identify the proteins acting at the plant-pathogen interface (Rep et al., 2002; Houterman et al., 2007). In the sap of infected tomato plants 14 small, Fol-derived proteins have been identified so far that are called SIX (Secreted In Xylem) proteins (Houterman et al., 2007; Lievens et al., 2009; Ma et al., 2010; Schmidt et al., 2013). Gene knockout studies revealed that Six1 (Avr3), Six3 (Avr2), Six5, and Six6 are required for full pathogenicity (Rep et al., 2004; Rep, 2005; Houterman et al., 2008, 2009; Gawehns et al., 2014) designating them as effectors *sensu strictu* (i.e., having a role in disease development).

A commonly observed response to pathogen attack is the production of so-called pathogenesis-related (PR) proteins by the plant. Since many of them possess antimicrobial activity, it is generally assumed that they play a role in defense (van Loon et al., 2006). In response to Fol infection the abundance of specific tomato proteins, including PR proteins, changes in the xylem sap (Rep et al., 2002; Houterman et al., 2007). Besides the appearance of these specific proteins, the abundance of others decreases, sometimes below the detection level resulting in an apparent disappearance. One protein whose abundance strongly decreases upon infection is XSP10, a 10 kDa protein with lipid-binding properties (Rep et al., 2003; Krasikov et al., 2011), which is required for full susceptibility of tomato to Fusarium wilt (Krasikov et al., 2011).

Since all effector knockout strains tested so far showed decreased pathogenicity, Fol effectors are predicted to have unique and non-redundant functions. Hence, each effector is expected to affect only a specific subset of plant responses. To test this hypothesis and to gain insight into these functions, we here set out to determine the changes in the xylem sap proteome following Fol infection using a label-free quantitative, large-scale proteomics approach. Thereto, xylem sap of tomato inoculated with either wild-type Fol or the available *AVR3, AVR2, SIX5*, or *SIX6* knockout strains and a newly created *SIX2* knockout was isolated, and analyzed using qLC-MS. To reveal changes in abundance of xylem sap proteins a bioinformatics pipeline was developed. Next, the xylem sap proteomes were compared with each other and with the proteome of either mock (water) or wild-type Fol inoculated plants. Surprisingly, not only a specific effect of Fol effectors on the xylem sap composition was revealed, but also a common one implying that effector proteins not only exert non-redundant activities but also have shared functions.

## Materials and methods

### Plant growth conditions

Tomato (*Solanum lycopersicum*) variety C32 was used for the Fol-disease assays and is susceptible to Fol races 2 (Fol007) (Kroon and Elergsma, 1993). Seedlings were transferred to pots 10 to 14-days-after-sowing and subsequently grown in a climatized greenhouse at 25°C, 65% relative humidity and a 16 h photoperiod.

### Generation of the *SIX2* knockout-constructs

To create the *SIX2* knockout construct, the DNA sequence from the position 1870–64 bp upstream of the *SIX2* open reading frame was PCR-amplified using primers with *Hind*III and *Xba*I linkers (5′-AAAAAGCTTGGACCGTACATAATGC TGCA-3′ and 5′-AAATCTAGAGCGGATAGAGATGAGATGA-3′) and inserted into the binary vector pRWh2 next to the hygromycin resistance cassette (Houterman et al., 2008). The sequence from 147 to 870 bp downstream of the *SIX2* stop codon was amplified using primers containing a *Kpn*I linker (5′- AAAGGTACCAAATCTATCCTCCAGGTT-3′ and 5′- AAAGGTACCATCATGCACGTTAATGAAAGTA-3′), and inserted on the other side of the hygromycin cassette.

### Transformation of fol, targeted knockout of *SIX2* and pathogenicity test

The transformation was performed using *Agrobacterium tumefaciens* mediated transformation (Takken et al., 2004) as described before (Gawehns et al., 2014). Briefly, spores of Fol007 (2 × 10^6^ spores/ml) and *A. tumefaciens* carrying the *SIX2* knockout-construct were co-cultivated on ME25 filters placed on IM plates (10 mM glucose, 10 mM K_2_HPO_4_, 10 mM KH_2_PO_4_, 2.5 mM NaCl, 4 mM (NH_4_)_2_SO_4_, 0.7 mM CaCl, 2 mM MgSO_4_, 9 μM FeSO_4_, 0.5% (w/v) glycerol, 5mM glucose, 1.5% Bacto-agar, 40 mM MES pH 5.3). Putative transformants were obtained after transfer of the filters on CDA (Czapek Dox Agar) containing cefotaxime. The hygromycin resistant monospores were PCR tested for successful transformation and deletion of *SIX2* using primer pairs 5′-TGGGCGGAATATATGA CCAT-3′/ 5′-GCATGTTTCTTCCTT GAACTCTC-3′ and 5′-TAGAGATCATGCTATATCTC-3′/5′-CGACACTCGCTTATCATGCA-3′.

To analyze pathogenicity of the *SIX2* knockout, 10-day-old seedlings were uprooted from the soil and for 5 min inoculated with a 5-day-old spore suspension (10^7^ spores/ml) of the *SIX2* knockout, the Fol007 wild-type or mock-treated (no spores) and subsequently potted (Wellmann, 1939). Disease symptoms of 15 plants/treatment were scored by means of plant weight and disease index (Gawehns et al., 2014) after 3 weeks. Significant differences between the treatments were tested using ANOVA (Fisher PLSD significant at 95%) and presented by the clustering they show in a dot plot.

### Plant inoculations and xylem sap collection

Spore suspensions (0.5 × 10^7^ spores/ml) were prepared from 5-day-old cultures of Fol007, Δ*AVR2*, Δ*AVR3*, Δ*SIX2*, Δ*SIX5*, and Δ*SIX6*. The soil, and part of the main root system, of 4-week-old C32 tomato plants was removed. Twenty-five plants per replicate were inoculated with Fol007, the knockout strains, or were mock-treated (water without spores) for 5 min and planted (Wellmann, 1939). Upon appearance of the disease symptoms (formation of air-roots, yellowing and wilting of the lower leaves), at approximately 14 dpi the xylem sap was collected as described (Rep et al., 2002; Krasikov et al., 2011). Briefly, plants were watered and the temperature was set at 22°C. The stems were cut just below the first real leaves and plants were placed horizontally to “bleed” for 6 h into a 12 ml polystyrene tube that was placed on ice. The collected xylem sap was stored at −20°C until further processing. Inoculation and xylem sap harvesting was independently repeated four times/experiment and the experiments were carried out in four subsequent weeks. Experiment 1 was done in the autumn of 2011, Experiment 2 early spring 2012.

### Sample preparation and mass spectrometry and label-free quantitative proteomics

Analysis of the samples was performed as described before (Schmidt et al., 2013). In summary, after removing the spores from the xylem sap by centrifugation, the sap of 25 plants was concentrated and trichloroacetic acid/aceton precipitated xylem sap samples (45 μg of protein) were boiled in SDS loading buffer (2% SDS, 10% glycerol, 50 mM Tris pH 6.8, 100 mM DTT, 0.05% bromphenol blue) and loaded on a SDS-polyacrylamide gel and shortly (1 cm) separated on SDS-PAGE (Mini-PROTEAN gel electrophoresis, Bio-Rad). Proteins were stained with Commassie PageBlue (ThermoFisher) revealing approximate equal amounts of proteins for each treatment. From each sample one slice containing all proteins was cut from the gel. Following *in-gel* digestion (Rep et al., 2002) the samples were dissolved into 50 μl 1 ml/l formic acid in water and the obtained peptides were analyzed by nanoLC-MS/MS. The samples were analyzed by injecting 18 μl sample over a 0.10 ^*^ 32 mm Magic C18AQ 200A 5 μm beads (Michrom Bioresources Inc., USA) pre-concentration column (prepared in-house) at a constant pressure of 270 bar (normally resulting in a flow of ca. 7 μl/min). Peptides were eluted from the pre-concentration column onto a 0.10 ^*^ 250 mm Magic C18AQ 200A 3 μm beads analytical column (prepared in-house) with an acetonitril gradient at a flow of 0.5 μl/min with a Proxeon EASY nanoLC. The gradient consisted of an increase from 8 to 33% acetonitril in water with 5 ml/l acetic acid in 50 min followed by a fast increase in the percentage acetonitril to 80% (with 20% water and 5 ml/l acetic acid in both the acetonitril and the water) in 3 min as a column cleaning step.

A P777 Upchurch microcross was positioned between the pre-concentration and analytical column. An electrospray potential of 3.5 kV was applied directly to the eluent via a stainless steel needle fitted into the waste line of the microcross. Full scan positive mode FTMS spectra were measured between m/z 380 and 1400 on a LTQ-Orbitrap XL (Thermo electron, San Jose, CA, USA) in the Orbitrap at high resolution (60,000). CID fragmented (Isolation width 2 m/z, 30% normalized collision energy, Activation Q 0.25 and activation time 15 ms) MSMS scans of the four most abundant 2 and 3+ charged peaks in the FTMS scan were recorded in data dependent mode in the linear trap (MSMS threshold = 5.000, 45 s exclusion duration for the selected m/z ± 25 ppm).

The MaxQuant software (Cox and Mann, 2008; Hubner et al., 2010) and MaxQuant 1.1.36 settings (Peng et al., 2012) were used to analyze the raw data generated by an LTQ-OrbitrapXL for protein identification and label-free quantification. The principles and algorithms underlying the label free quantification (LFQ) method is described elsewhere (Cox et al., 2014). Default settings for the Andromeda search engine were used (Cox et al., 2011) including 1% FDR cutoff values for peptides and proteins, except that extra variable modifications were set for de-amidation of N and Q. Identification of the tomato proteins was based on the SGN tomato protein database ITAG2 version 3 (34,727 entries) (ftp://ftp.solgenomics.net/../../proteins/protein_predictions_from_unigenes/single_species_assemblies/Solanum_lycopersicum/), while for fungal proteins the database from the Fusarium Comparative Genome website (http://www.broadinstitute.org/annotation/genome/fusarium_group/MultiHome.html) was used. The latter database (total 17,652 entries) was supplemented by adding the sequences of Six proteins that are not annotated in the public database. A “contaminant” database (59 entries) was used to identify proteins such as trypsin and human keratins (Peng et al., 2012). The “label-free quantification” as well as the “match between runs” (set to 2 min) options were enabled. De-amidated peptides were allowed to be used for protein quantification and all other quantification settings were kept default. The mass spectrometry proteomics data have been deposited to the ProteomeXchange Consortium (Vizcaíno et al., 2013) via the PRIDE partner repository with the dataset identifier PXD003010.

Filtering and further bioinformatics analysis of the MaxQuant/Andromeda workflow output and the analysis of the abundances of the identified proteins were performed with the Perseus 1.3.0.4 module (available at the MaxQuant suite). Accepted proteins had at least 2 identified peptides of which at least one should be unique and at least one should be unmodified. Reversed hits were deleted from the MaxQuant result table. LFQ values were log10 transformed.

### Data processing

For proteins, which were not detected in one sample but were present in one of the other samples of the same experiment, fixed log10 LFQ values (6.0 for Experiment 1 and 5.5 for Experiment 2) were imputed. Proteins that were quantified by at least 2 peptides in at least 3 out of 4 biological replicates were annotated as “Present” (P), those quantified in 1 or 2 biological replicates were referred to as “Marginal” (M) while the others were called “Absent” (A). To allow for making statements about significance and fold change all proteins (P+M) were kept in the datasets of each experiment for follow-up analyses. Data handling was performed mostly in the R-2.15.1 program (http://www.r-project.org/) together with the bioconductor (http://www.bioconductor.org/) packages limma and maanova. As technical differences between Experiments 1 and 2 were present, these datasets were handled independently from one another on the level of data processing and low-level analysis.

Subsets of plant specific proteins were generated by removing the fungal and contaminating non-plant proteins. Next, the log10 LFQ values were median normalized over the samples per experiment to obtain more comparable data distributions. PCA plots of these normalized data were then generated. For each dataset the following mixed linear model was fitted per protein on all samples (Wolfinger et al., 2001; Cui and Churchill, 2003):

$$\begin{array}{l} y=\mu +T+W+\varepsilon \end{array}$$

Where μ captures the average protein abundance, *T* captures the variation for the different treatments, *W* is a random factor that captures the variation for the different weeks in which the experiments were performed and ε is the residual error for each individual sample.

For hypothesis testing a permutation-based F1 test (2000 permutations) was applied to the values for treatment from the model fit with all pairwise comparisons of each samples to one another (Cui and Churchill, 2003). False discovery rate (FDR) correction was performed using *q*-values (Storey and Tibshirani, 2003) over all tests from the different comparisons combined. This is an approach that is specifically designed to correct for false discovery in multiple-hypothesis testing with features that are represented in a genome. FDR adjusted p-values < 0.1 were considered as statistically significant (Supplemental Table 1). This workflow was adapted from Ting et al. (2009).

### Gene ontology (GO) analysis

To batch-classify the xylem sap proteins into functional plant categories the software Mercator (http://mapman.gabipd.org) was used. Mercator uses MapMan categories, which categorize proteins in metabolic pathways and enzyme functions. For the analysis three sequence classifications were performed: Blast searches against Arabidopsis TAIR10, plant proteins from swiss-prot and UniRef90, RPS-Blast searches against cdd and KOG and an InterPro scan. Default settings were used to analyze the xylem sap proteins. The obtained MapMan bin-codes were sorted manually into 10 gene ontology classes as shown in Supplemental Table 2 and every protein was assigned a single bin-code.

## Results

### Characterization of the tomato xylem sap proteome upon fol infection

**Figure 1:**
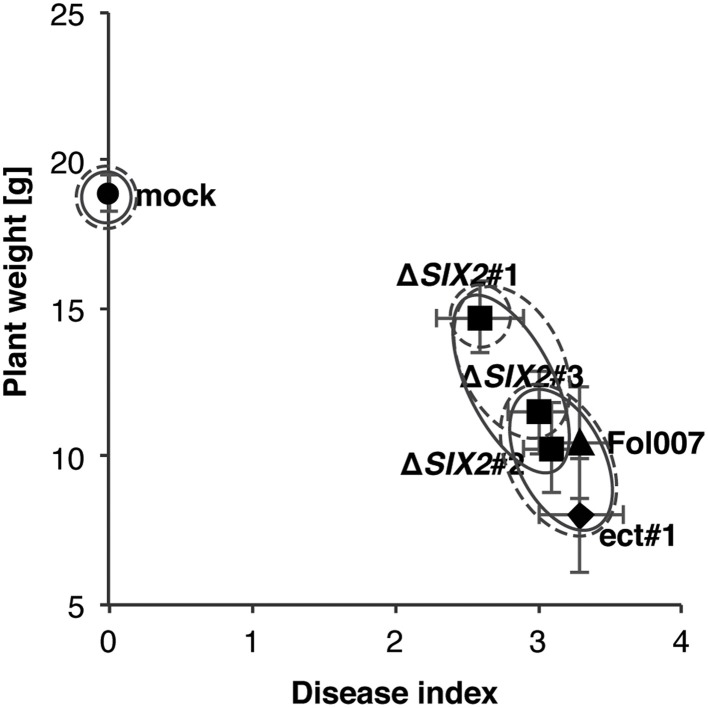
**One *SIX2* knockout is reduced in virulence**. Susceptible tomato seedlings were inoculated with wild-type Fol (Fol007) or strains in which *SIX2* was deleted (Δ*SIX2*#1-3). As controls mock-inoculated plants were used (mock) or one transformant (ect#1), in which the *SIX2* deletion construct was integrated ectopically. Average plant weight of 15 plants was plotted against the average disease index of the same plants. In Δ*SIX2*#1 pathogenicity was impaired as shown by the increased plant weight as compared to infection with Fol007 and the ectopic transformant. Error bars represent the standard error. Clustering is based on an ANOVA (Fisher PLSD significant at 95%) using either the disease index (solid line) or plant weight (dashed line).

To determine the xylem sap proteome composition and the quantitative changes therein due to specific effector proteins, we collected xylem sap from tomato plants inoculated with *F. oxysporum* isolate Fol007 and different effector knockout strains in the Fol007 background. Besides the previously described *AVR2, AVR3, SIX5*, and *SIX6* effector knockout strains (Rep et al., 2005; Houterman et al., 2009; Schmidt et al., 2013; Gawehns et al., 2014) also an *SIX2* knockout strain was included. Three *SIX2* knockout strains were made, of which only one strain (Δ*SIX2*#1) showed reduced virulence in a seedlings bioassay, resulting in increased plant weight and a reduction of the disease index (i.e., vessel browning, stunting and wilting, Gawehns et al., 2014); the other two knockout strains and a reference strain with the *SIX2* knockout construct ectopically inserted into the genome behaved like wild-type (Figure 1). Transformant Δ*SIX2*#1 was used for further analysis in this study.

Two experiments were performed in a climatized greenhouse, one in autumn (Experiment 1) and one in spring (Experiment 2). Besides mock and Fol007 inoculations the Δ*AVR2* and Δ*SIX5* strains were included in Experiment 1, while Δ*AVR3*, Δ*SIX2*, and Δ*SIX6* were used in Experiment 2 (see below). Roots of 4-week-old plants were inoculated and xylem sap was collected approximately 14-days-post-inoculation (dpi) after early disease symptoms developed (formation of air-roots, yellowing and wilting of the lower leaves) (Rep et al., 2002). The procedure resulting in the identification and quantification of the xylem sap proteome is schematically depicted in Figure S1. Briefly, the xylem sap of the 25 plants/treatment was pooled, resulting in a typical yield of 15–70 ml. The collected xylem sap proteins were concentrated, separated (SDS-polyacrylamide gel) and subjected to *in-gel* tryptic digestion. Identification of the released peptides was done using nLC-MS/MS mass spectrometry followed by MaxQuant analysis to identify and quantify the progenitor proteins (Supplemental Tables 3, 4) For reliable identification only proteins were included that matched with at least two peptides of which at least one was unique.

**Figure 2:**
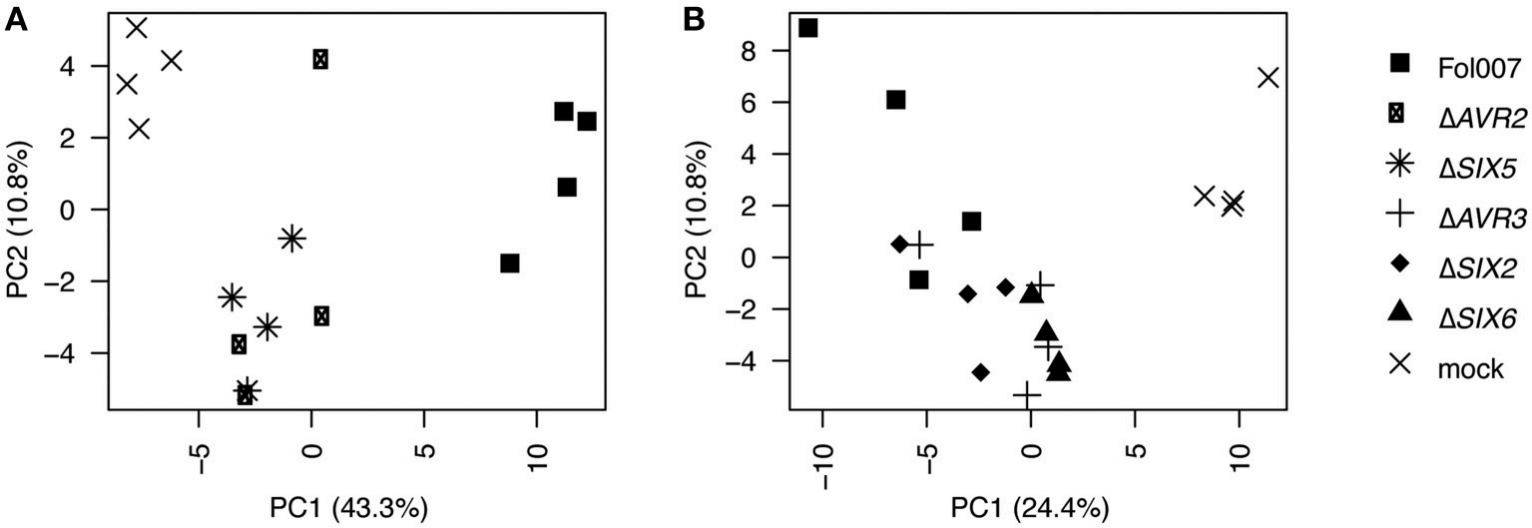
**Technical replicates of each treatment cluster along the x-axis**. PCA plot based on the normalized proteome data. PC1 is plotted on the x-axis, PC2 is plotted on the y-axis. The PCA analysis was performed with all 16 individual treatments from data set 1 **(A)** and the 20 treatments from data set 2 **(B)**.

In some cases, proteins were not found consistently among all replicates of a treatment. To allow inclusion of these proteins in subsequent analyses the following definitions were used: “Absent” (A) for proteins that were not identified in any of the four replicates, “Marginal” (M) for proteins found in only one or two replicates or “Present” (P) when found in three or all four replicates. A protein was considered “found” when either labeled “Marginal” or “Present.” To allow for statistical testing of significant differences between the xylem sap proteins of mock and Fol-inoculated plants the LFQ intensity data, with imputed values for “Absent” proteins, were normalized using log10 transformation and a median scaling of the protein abundance data distributions over the replicates of all treatments for each experiment separately (Figure S2). Figure 2 shows a principal component analysis (PCA) plot, generated from these normalized data, representing all treatments and replicates per experiment. The distribution of all replicates of each treatment was found to be comparable along the PC1-axis. This even distribution indicates that each replicate was influenced by similar effects allowing the replicates of each treatment to be analyzed together. In Experiments 1 and 2, respectively, a total of 343 and 292 plant proteins (Supplemental Table 1), 43 and 19 Fol proteins and 7 contaminating proteins (e.g., trypsin, keratin etc.) were identified and quantified. Analysis of the Fol-encoded proteins has been reported elsewhere (Schmidt et al., 2013); here we solely focus on the plant proteome.

To assess the purity of the identified xylem sap the proteome was analyzed for the presence of typical proteins from xylem and absence of phloem-derived and intracellular proteins. Typical xylem sap proteins that are conserved among different plant species, like peroxidases, glycine-rich proteins, serine and aspartyl proteases, chitinases and lipid transfer protein-like polypeptides (Buhtz et al., 2004) were present in both experimental sets. In addition 16 intracellular proteins were identified, including enzymes like Ribose-5-phosphate isomerase, Triose-phosphate-isomerase, Fructose-bisphosphate-aldolase and Fructose-1,6-bisphosphatase catalyzing reactions in gluconeogenesis, glycolysis and the Calvin cycle. Also a Polyphenoloxidase was identified, which is a characteristic protein for phloem sap (Dafoe and Constabel, 2009). Other typical phloem proteins, like PP1 and WIN4 (Dafoe and Constabel, 2009; Subramanian et al., 2009), or the intracellular Ribulose-1,5-bisphosphate carboxylase however, were absent. Notably, of the 16 intracellular proteins only one, a ferredoxin-related protein (Solyc07g063740.2.1), was consistently found in all 4 replicates in Experiments 1 and 2. Only 3 of the 15 others were found in both experiments and their presence where mostly scored as marginal/absent in Experiment 2. Together, this suggests some, but minimal contamination of phloem sap in the xylem sap preparation.

### The tomato xylem sap proteome composition changes distinctively after fol infection

**Figure 3:**
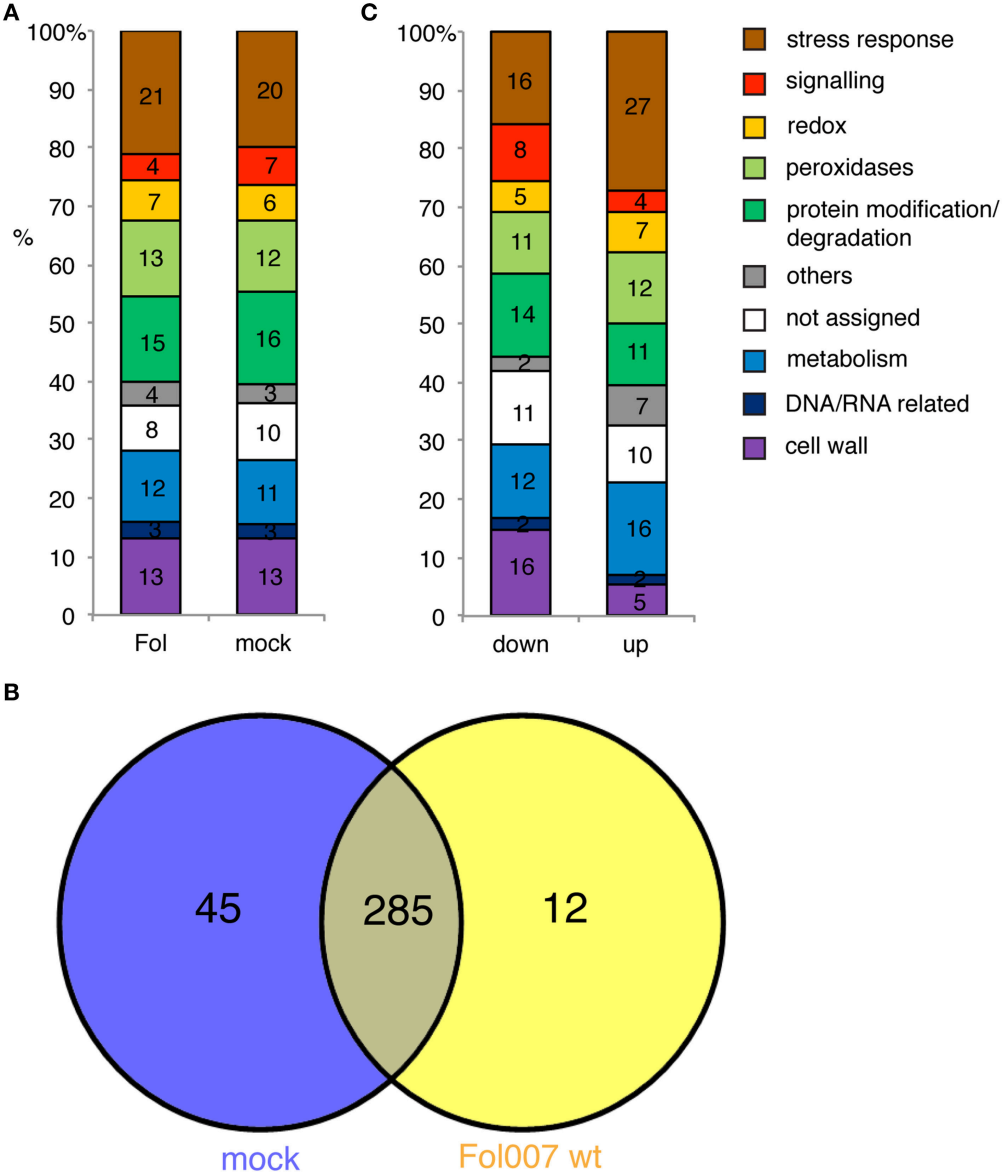
**The identity of the xylem sap proteome in mock-treated and Fol-inoculated plants is similar. (A)** Bar chart showing the percentage of xylem sap proteins per GO category for Fol-inoculated and mock-inoculated plants. **(B)** Venn-diagram representing the number of overlapping and individual proteins of the mock and Fol proteome. **(C)** Bar chart depicting all different accumulated proteins (DAPs) in the Fol vs. mock contrast depending on whether the protein abundance was decreased (down) or increased (up).

To assess the effect Fol has on the xylem sap proteome composition, the identities of the plant proteins present in the Fol- and mock-treated plants of Experiment 1 were compared. The data obtained in Experiment 1 were preferred over the data set of Experiment 2 because of the larger number of proteins identified (342 vs. 292). In Experiment 1 297 and 330 tomato proteins were identified in Fol- and mock-treated plants, respectively. The possible functions of the xylem sap proteins were determined by Gene Ontology (GO) annotation using the plant optimized and homology-based annotation software MapMan (Klie and Nikoloski, 2012; Lohse et al., 2014). The obtained bin-codes were classified into 10 categories: “cell wall,” “metabolism,” “stress responses,” “redox,” “peroxidases,” “DNA/RNA,” “protein modification,” “signaling,” “others” including mostly photosynthesis and development related proteins and “not assigned” (Supplemental Table 2). The largest category is formed by proteins belonging to “stress responses” with 21 and 20% of the total proteins found in the Fol and mock proteome respectively (Figure 3A). This is followed by the categories “protein modification and degradation,” “cell wall,” “peroxidases,” and “metabolism.” The categories containing the lowest number of proteins are “others,” “redox,” and “signaling” The latter group is most distinct in relative size between Fol and mock. In general, however, the size and composition of the categories are similar for both treatments.

**Figure 4:**
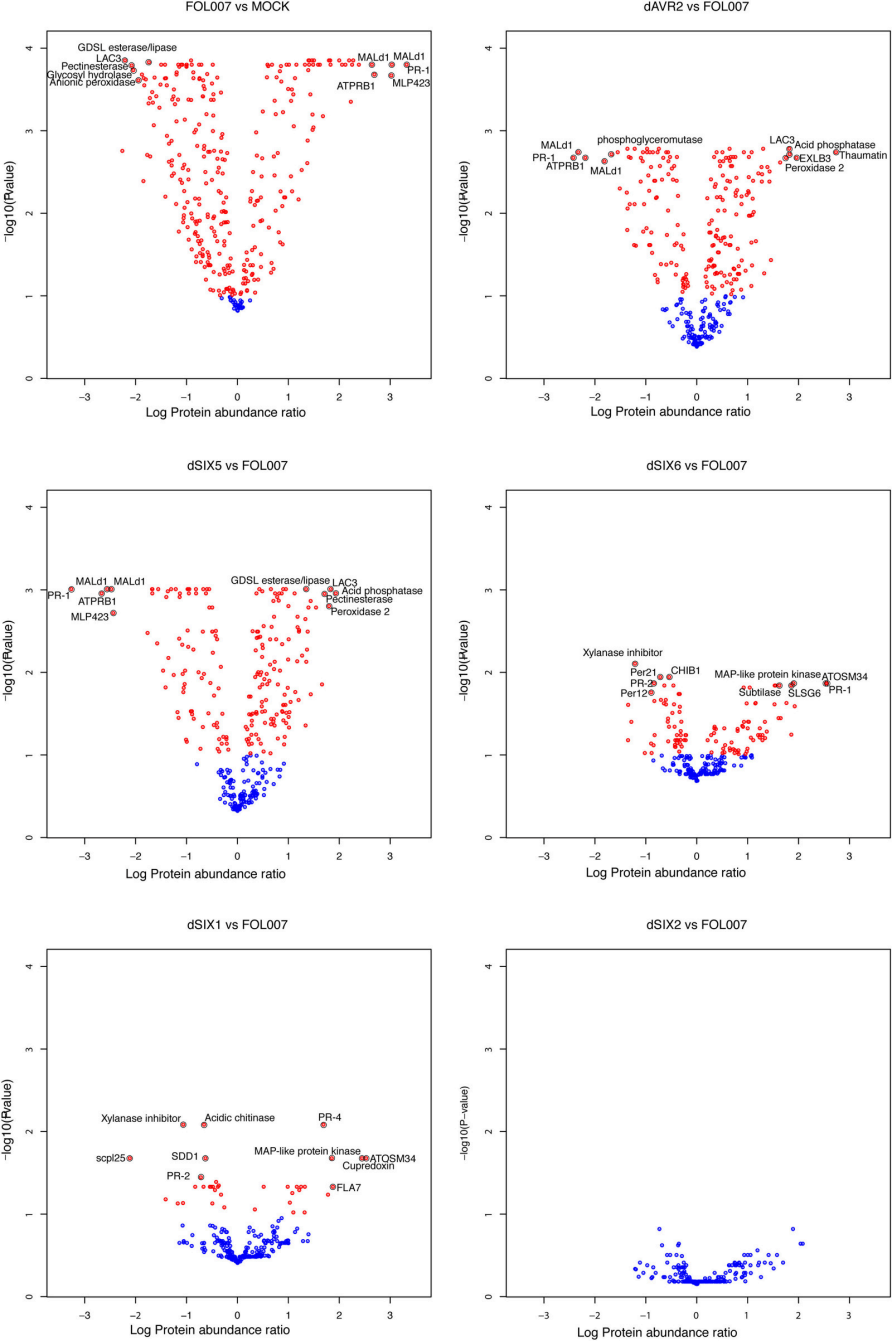
**The number of DAPs differs substantially between the different contrasts that were tested**. Volcano plots showing the relation between -log10 False Discovery Rate-corrected *P*-values and log10 protein abundance ratios, based on the group average per treatment from the linear model fit. Blue circles indicate proteins that are not differential while red circles were annotated as DAPs. The black encircled DAPs, with names included, represent the top-10 combinations of lowest P-value and strongest up- and down-regulated proteins of Fol compared to Mock (top left), dAVR2 compared to Fol (top right), dSix5 compared to Fol (center left), dSix6 compared to Fol (center right), dAvr3 compared to Fol (bottom left) and dSix2 compared to Fol (bottom right). Names of the proteins can be found in **Table 2**.

Only 12 proteins were identified exclusively in Fol-treated plants, while 45 were solely found in the mock. In total, 285 plant proteins were identified in both Fol and mock (Figure 3B). A data pipeline was developed to identify proteins whose abundance changes significantly following infection. Thereto, a mixed linear model was fitted per protein on all samples to correct the data for the non-treatment related noise caused by sampling the replicates in four different weeks, and to estimate the effect of the different treatments. Next, the contrasts between xylem sap proteins in mock and Fol-inoculated plants were tested for significant differences using 2000 permutations to relax restrictions of normality. Subsequently, a correction for false discovery rate was done (*p* < 0.1). Proteins exhibiting a significantly altered abundance after Fol inoculation compared to the mock treatment were called DAPs, for Differentially Accumulated Proteins (DAPs). This analysis revealed that of the 285 plant proteins common to both datasets, the abundance of 27 proteins was unaltered (Figure 4, top left panel, blue circles and Supplemental Table 5), whereas that of the other 258 proteins varied significantly (*p* < 0.1) between Fol and mock treated plants (Figure 4, top left panel, red circles). The abundance of 156 proteins was decreased (Figure 4, red circles left of the “0” axis) and that of 102 proteins increased upon Fol inoculation (Figure 4, red circles right of the “0” axis and Supplemental Table 1). In total, 315 DAPs were identified; 258 are present in both Fol and mock samples, 12 are unique for Fol treatment and 45 are unique for the mock. Figure 3C shows the distribution of the 315 DAPs over the GO categories. We separated them in two groups: the “up” group consisting of emerging proteins and those whose abundance increases upon Fol treatment, and the “down” group contains all DAPs with reduced amounts and proteins undetected after Fol treatment. Most proteins in the “down” group belong to the categories “cell wall” or “stress responses.” These categories each account for respectively 15 and 16% of the total dataset. The category “protein modification and degradation” covers 14%. Of the “up” DAPs the category “cell wall” is depleted and represents only 5% of the proteins. The same pattern is observed regardless whether the 45 mock-unique proteins were included in the analysis or not (data not shown). The “up” DAPs were especially enriched for the category “stress responses,” harboring 27% of the total number of identified proteins.

In summary, the abundance of 92% of the tomato proteins present in the xylem sap changes after infection while that of only 8% remained unaltered. Of the altered proteins 6% was identified solely after Fol inoculation. Hence, upon infection the overall xylem sap proteome composition—in terms of GO categories—remains relatively constant, but the abundance of specific proteins changes distinctively. Generally, the abundance of proteins related to stress-responses increased while those having cell wall-related activities became less abundant.

### Data from different experiments can be combined

**Figure 5:**
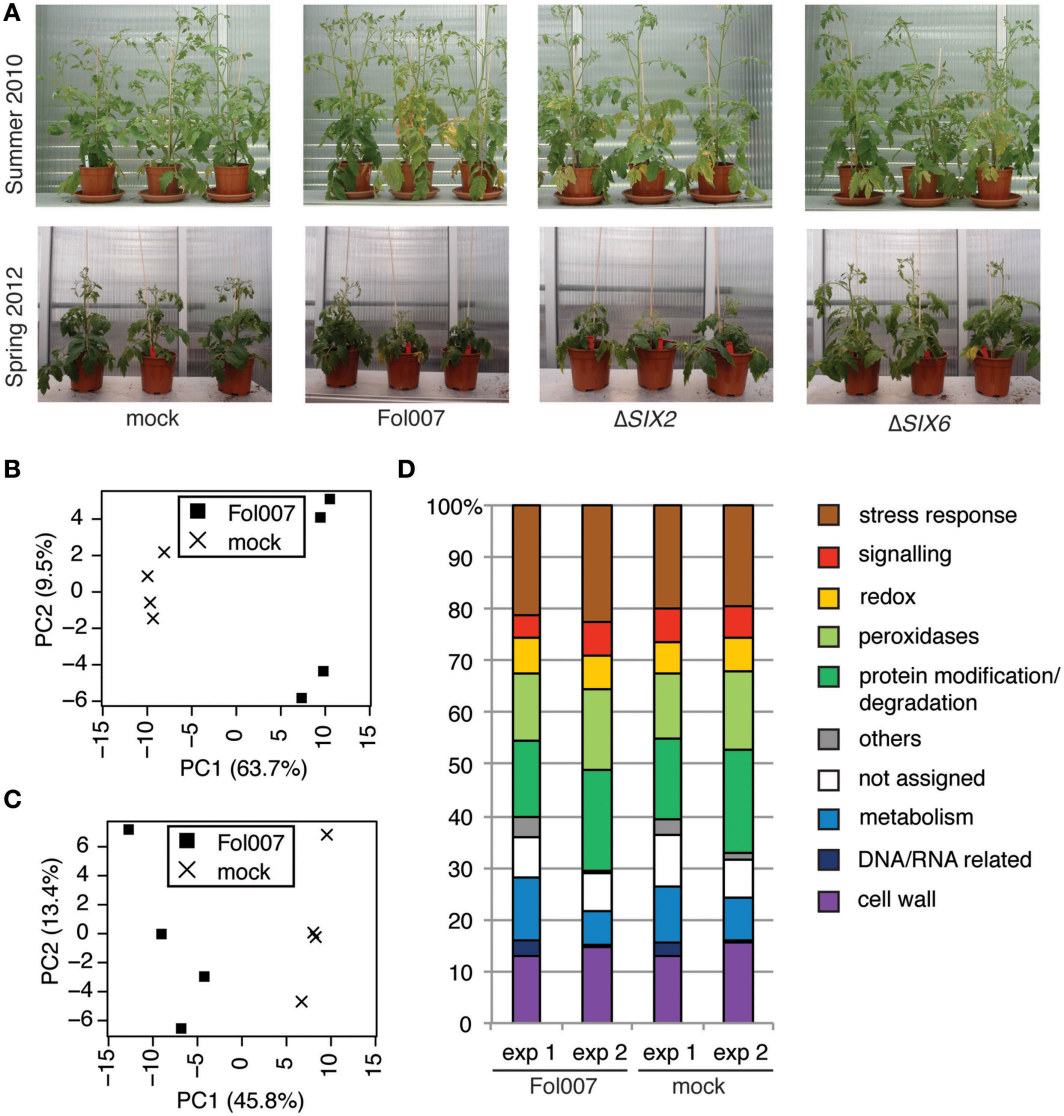
**The proteome of xylem sap harvest performed in different seasons is comparable on data- and biological-level. (A)** Photographs taken from two different bioassays performed in either summer 2010 or spring 2012, when clear disease symptoms developed (2–3 weeks after inoculation). Approximately the same magnification was used. Four-week-old plants were mock-treated or inoculated with Fol007, the *SIX6* knockout (Δ*SIX6*) or the *SIX2* knockout (Δ*SIX2*). Plants from summer 2010 were longer and disease symptoms included mainly wilting and yellowing of the leaves. In spring 2012 diseased plants were recognized by epinasty and reduced plant size. **(B,C)** PCA plot based on the normalized data. PC1 is plotted on the x-axis, PC2 is plotted on the y-axis. The PCA analysis was performed with the proteome data obtained for Fol- or mock-treated plants from data set 1 **(B)** or from data set 2 **(C)**. **(D)** Bar chart showing the percentage of xylem sap proteins per GO category for the Fol and mock treatments of data set 1 and data set 2.

Also in conditioned greenhouses the symptoms of Fusarium wilt disease are subject to seasonal influences. Figure 5A shows plants treated with either water or with one of the three Fol strains (Fol007, Δ*SIX2*, and Δ*SIX6*) in two different seasons (summer 2010 and early spring 2012). Inoculation in spring typically results in shorter plants, later onset of wilting symptoms, and a stronger epinastic response as compared to bioassays performed in summer. Nevertheless, the same relative effect of the inoculations on disease symptoms were observed: plants inoculated with Fol007 or Δ*SIX2* showed the severest disease symptoms while inoculation with Δ*SIX6* or mock treatment caused mild or no symptoms.

To assess the overall quality and comparability of the data sets obtained in Experiments 1 and 2, PCA analysis of the normalized LFQ values of all plant proteins was performed for the mock- and Fol-treated plants. The PCA plots for both data set 1 (Figure 5B) and data set 2 (Figure 5C) show a clear separation of the mock treatment from the Fol treatment on the PC1 axis. Hence, the biological effects were stronger than the technical effects and therefore we considered the quality of the data to be sufficient for further analysis. In addition, a PCA based on all proteins found in the four datasets (Figure S3) shows that, although the technical and biological variation explains most of the variance in the data (PC1), the Fol007 and mock clearly separate on the PC2 axis for both experiments. To determine the similarity between both sets on the biological level, a GO analysis was performed using the xylem sap proteome of either mock- or Fol-inoculated plants. The results of this analysis are plotted in a bar chart (Figure 5D). The largest group of proteins in all treatments belonged to the category “stress response,” followed by the categories “protein modification and degradation,” “cell wall,” “peroxidases,” and “metabolism.” The categories “signaling,” “redox,” “DNA and RNA related,” “others,” and “not assigned” were least represented. In the data set of Experiment 1 relatively more metabolism-related proteins were identified, whereas in Experiment 2 relatively more proteins with a function in protein modification and degradation were detected. The abundance of proteins assigned to “others” and “DNA and RNA related” was relatively high in Experiment 1 compared to Experiment 2.

A correlation analysis was performed with the data from Experiments 1 and 2 (Figure S4) to analyze the comparability of the data. In the scatter plot the data distribute on a linear line through 0 by trend. The Pearson correlation coefficient of this analysis is 0.6, which indicates a positive correlation between the data sets. In conclusion, although differences between the severity of disease symptoms were observed between Experiments 1 and 2 both data sets are similar regarding overall biological differences and data-quality, allowing direct comparison of the DAPs identified in both sets for further analysis.

### Avr2, Avr3, Six5, and Six6 have specific *and* common effects on the xylem sap proteome composition

Next, we examined whether reduced pathogenicity can be correlated to a specific change in the xylem sap proteome composition. Comparing the DAPs identified in Experiments 1 and 2 revealed changes in the xylem sap proteome after inoculation with the five Fol effector knockout strains. To analyze the differences in the xylem sap proteome in relation to the absence of specific effectors, the DAPs for each knockout were determined using a *p* < 0.1. The contrast between the *AVR2* knockout and Fol wild-type revealed 209 DAPs; 37 DAPs were found for Δ*AVR3*, 0 for Δ*SIX2*, 206 for Δ*SIX5* and 115 for Δ*SIX6* (Figure 4). The absence of DAPs between Δ*SIX2* and Fol wild-type is noticeable and corroborates the chosen settings for FDR and p-values as being sufficiently stringent. Because the xylem proteome composition after inoculation with the Δ*SIX2* knockout was identical to that of the wild-type Fol (Figure 4, bottom right panel) these contrasts are not depicted separately in the subsequent analyses. Note that also the disease symptoms of the Δ*SIX2* Fol inoculation of the 4-week-old plants were indistinguishable from that of the wild-type (Figure 5A).

**Figure 6:**
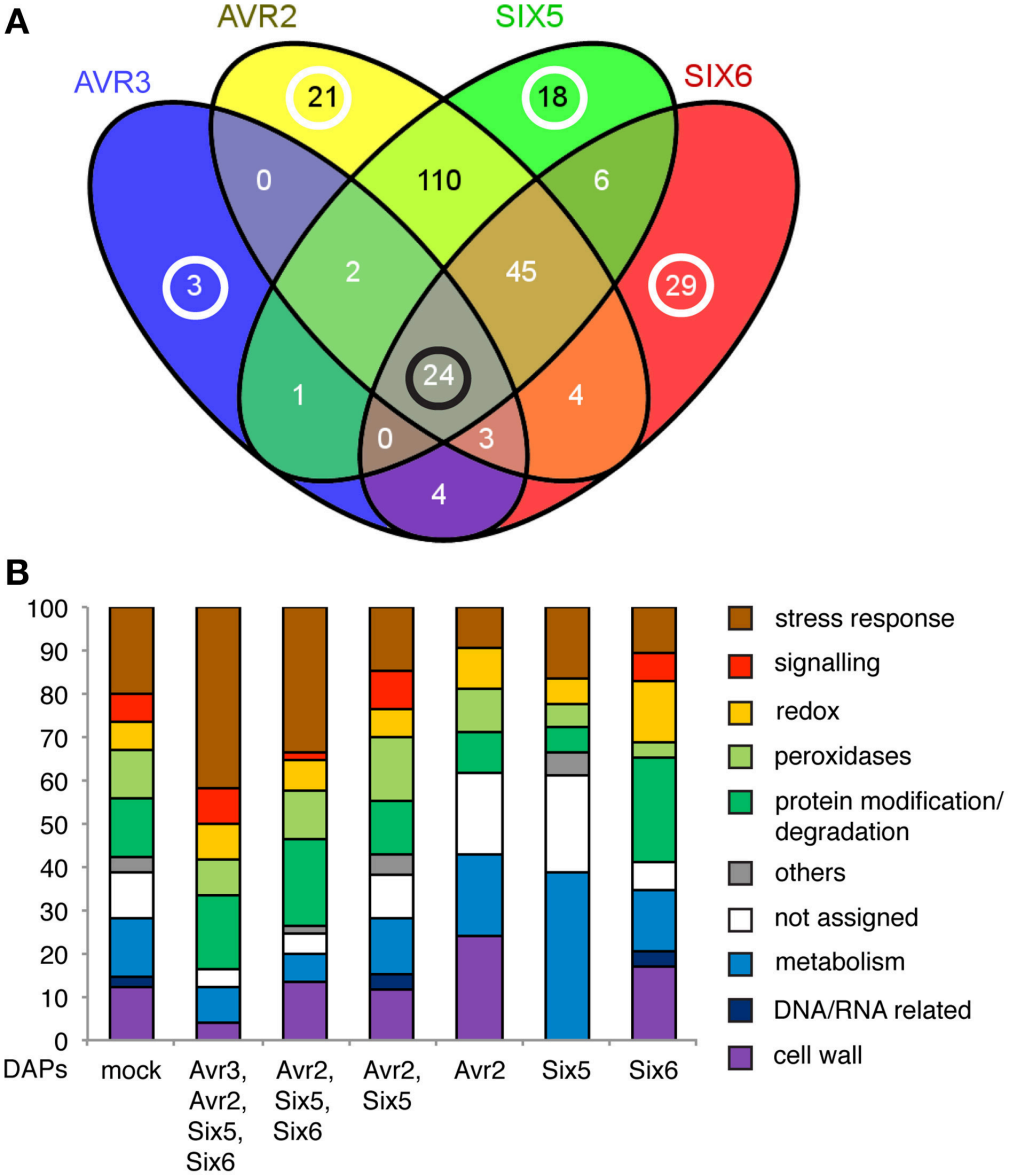
**Effector knockouts have common and specific DAPs. (A)** Venn-diagram showing the overlap of DAPs in the contrasts between the *AVR3, AVR2, SIX5, SIX6* knockouts with Fol007. Black circle: number of DAPs shared by all effector knockouts, white circles: number of effector knockout specific DAPs. **(B)** Bar chart depicting the percentages per GO category for all DAPs in the contrast between Fol007 and mock (mock) and for all groups of common [overlap in **(A)**: Avr2, Avr3, Six5, Six6, or Avr2, Six5, Six6, or Avr2, Six5] or specific DAPs (Avr2 or Six5 or Six6) in the contrasts between effector knockouts and Fol007. Groups of DAPs in the Venn-diagram containing less than 10 proteins are not shown.

The overlap between the DAPs from different knockouts is depicted in Figure 6A. In line with their combined function in the plant (Ma et al., 2015), the largest number of DAPs (110) is shared between the *AVR2* and *SIX5* knockouts. Twenty-four DAPs are shared by the *AVR3, AVR2, SIX5*, and *SIX6* knockout strains (Figure 6A). Ten of those DAPs belong to the category “stress response” and are mostly PR proteins (**Table 2** and Figure 6B). Also four proteases from the category “protein modification and degradation” are present in this set. In all xylem sap samples from plants treated with one of the above four effector-knockout strains the abundance of those proteases was decreased compared to the Fol wild-type. Also from each of the categories “signaling,” “redox,” “peroxidases,” and “metabolism,” two proteins belonged to the common DAPs. These 24 DAPs are likely virulence-associated DAPs as their abundance is affected by all Fol strains that show a reduction in virulence and nearly half of them have a function in the stress response (**Table 2**).

Next, DAPs were identified whose abundance uniquely alters in xylem sap isolated from plants infected with a specific effector knockout strain. The gene ontology analysis of the specific DAP-sets showed a profile distinct from that of the common DAPs (Figure 6B). The largest change was observed in the percentage of proteins belonging to “stress responses” covering 42% for the common DAPs and, e.g., only 10% for the *AVR2*-specific DAPs. The profiles of the specific DAPs differed significantly from each other, as each profile was unique for a specific knockout.

In more detail, six DAPs were found in the xylem sap from plants inoculated with the Fol *AVR3* knockout when compared to the Fol wild-type (Figure 6A). These DAPs are a Kunitz-type proteinase inhibitor (Solyc03g098710.1.1), which is increased in abundance in the xylem sap, a polygalacturonase-like protein (Solyc09g075460.1.1), two serine carboxypeptidases (Solyc06g083040.1.1 and Solyc02g088820.1.1), one superoxide dismutase (SOD) (Solyc01g067740.1.1) and one PR-2 (Solyc04g080260.1.1 a Glucan endo-1 3-beta-glucosidase) protein, of which abundances were all decreased in the absence of Avr3. Three of these DAPs are unique to the *AVR3* knockout (Solyc09g075460.1.1, Solyc06g083040.1.1, and Solyc03g098710.1.1) while the other three (Solyc01g067740.1.1, Solyc04g080260.1.1, and Solyc02g088820.1.1) exerted an Avr3-specific change in their abundance. For instance PR-2 abundance (Solyc04g080260.1.1) is decreased in Δ*AVR3* while it is increased in the Δ*SIX5* strain as compared to xylem sap of Fol wild-type infected plants. The two other DAPs are reduced in abundance in the Avr3 knockout while their abundance is increased in both the Avr2 and Six5 knockouts, bringing the total number of Avr3-specific DAPs to six.

In the xylem sap of tomato plants that were inoculated with the *AVR2* knockout strain 21 unique DAPs were found (Figure 6A). Also one DAP, a Endo-1 4-beta-xylanase (Solyc11g040330.1.1), is found that is shared with the *SIX6* knockout but it abundance is increased rather than decreased in the Δ*AVR2* strain. Those proteins represent all GO categories, except “signaling,” “others,” and “DNA/RNA related.” Notably, only 10% of these DAPs belong to “stress responses,” 24% to “cell wall” and 19% to “others” (Figure 6B). Table 1 shows all 22 Δ*AVR2*-specific DAPs with either increased or decreased abundance. Next to common DAPs like PR proteins, peroxidases, proteinases and different hydrolases, a Translationally Controlled Tumor Protein (TCTP) homolog and a Group II intron splicing factor CRS1-like protein was found. The first one showed a decrease in abundance the latter an increase.

**Table 1:** **List of proteins, whose abundance changes specifically upon infection with a *SIX* knockout as compared to wild-type Fol**.

**ID**	**GO**	**Functional description**	**u/d**	**log10 LFQ**	**log10 FC Six vs. Fol007**	**adjPval Six vs. Fol007**
**Avr3**						
Solyc09g075460.1.1	cw	Polygalacturonase-like protein-like	d	7.5	−1.1	0.07
Solyc06g083040.1.1	p	Serine carboxypeptidase 1	d	7.6	−0.3	0.06
Solyc02g088820.1.1	p	Serine carboxypeptidase K10B2.2	d	5.9	−2.1	0.02
Solyc01g067740.1.1	r	Superoxide dismutase	d	7.8	−1.2	0.07
Solyc03g098710.1.1	sr	Kunitz-type proteinase inhibitor A4 (Fragment)	u	6.7	1.1	0.10
Solyc04g080260.1.1	sr	Glucan endo-1 3-beta-glucosidase (PR-2)	d	9.0	−0.5	0.05
**Avr2**						
Solyc08g081480.1.1	cw	Polygalacturonase-like protein	d	8.8	−0.2	0.08
Solyc04g007160.1.1	cw	Alpha-glucosidase	u	6.7	0.7	0.09
Solyc04g077190.1.1	cw	Endo-1 4-beta-xylanase	u	9.1	0.3	0.04
Solyc05g009470.1.1	cw	Alpha-glucosidase	u	8.5	0.7	0.10
Solyc07g053540.1.1	cw	Fasciclin-like arabinogalactan protein	u	8.1	0.7	0.06
Solyc07g065110.1.1	m	Protease inhibitor/seed storage/lipid transfer protein family protein	d	7.8	−0.3	0.07
Solyc01g068380.1.1	m	Purple acid phosphatase	u	6.8	0.7	0.06
Solyc05g013720.1.1	m	Alpha-galactosidase	u	8.3	0.5	0.05
Solyc06g050130.1.1	m	Alpha-galactosidase-like protein	u	6.3	0.3	0.09
Solyc01g099770.1.1	na	Translationally-controlled tumor protein homolog	d	7.9	−0.3	0.06
Solyc08g075390.1.1	na	Isopentenyl-diphosphate delta-isomerase family protein	d	7.1	−0.6	0.04
Solyc12g014440.1.1	na	BNR/Asp-box repeat protein	d	6.0	−0.8	0.07
Solyc02g087250.1.1	na	Group II intron splicing factor CRS1-like	u	7.9	0.8	0.06
Solyc11g040330.1.1	na	Endo-1 4-beta-xylanase	u	8.1	0.4	0.05
Solyc02g092670.1.1	p	Subtilisin-like protease	d	9.7	−0.2	0.06
Solyc09g098340.1.1	p	Aspartic proteinase-like protein	d	8.2	−0.2	0.07
Solyc05g046010.1.1	po	Peroxidase	d	8.2	−0.2	0.08
Solyc02g079510.1.1	po	Peroxidase	u	7.8	1.3	0.05
Solyc03g083900.1.1	r	Laccase-22	d	8.2	−0.4	0.02
Solyc10g007070.1.1	r	CT099	u	8.1	0.2	0.08
Solyc12g008580.1.1	sr	Glucan endo-1 3-beta-glucosidase	d	8.1	−0.3	0.07
Solyc12g056390.1.1	sr	Thaumatin-like protein	u	8.8	2.7	0.00
**Six5**						
Solyc06g073190.1.1	m	Fructokinase-like	d	7.8	−0.4	0.08
Solyc09g009020.1.1	m	Enolase	d	7.6	−0.6	0.03
Solyc01g087610.1.1	m	Alpha-N-acetylglucosaminidase	u	8.0	0.3	0.07
Solyc02g070070.1.1	m	FAD-binding domain-containing protein	u	6.5	0.5	0.07
Solyc08g067500.1.1	m	Non-specific lipid-transfer protein	u	8.3	1.0	0.05
Solyc10g075100.1.1	m	Non-specific lipid-transfer protein	u	8.4	0.3	0.03
Solyc11g066290.1.1	m	Icc family phosphohydrolase	u	8.8	0.7	0.04
Solyc04g045340.1.1	na	Phosphoglucomutase	d	6.3	−1.0	0.07
Solyc12g014270.1.1	na	Peptide-N4-(N-acetyl-beta-glucosaminyl)asparagine amidase A	d	6.4	−1.1	0.00
Solyc04g054980.1.1	na	Lipoxygenase homology domain-containing protein	u	6.9	0.9	0.03
Solyc08g074620.1.1	na	Polyphenol oxidase	u	7.4	0.9	0.07
Solyc10g074820.1.1	na	Unknown Protein	u	7.7	1.3	0.04
Solyc05g052600.1.1	o	Fructose-1 6-bisphosphatase class 1	d	6.6	−1.2	0.04
Solyc03g123900.1.1	p	Mannosyl-oligosaccharide 1 2-alpha-mannosidase	u	7.8	0.5	0.09
Solyc12g099160.1.1	p	Serine carboxypeptidase K10B2.2	u	8.8	0.3	0.04
Solyc05g046020.1.1	po	Peroxidase	u	8.0	1.1	0.02
Solyc09g007520.1.1	po	Peroxidase	u	9.5	0.3	0.10
Solyc08g079090.1.1	r	Laccase-22	u	8.5	0.3	0.10
Solyc01g108840.1.1	s	Receptor-like kinase	u	8.2	0.2	0.06
Solyc02g061770.1.1	sr	Endochitinase	d	9.3	−0.4	0.06
Solyc04g007910.1.1	sr	Glucan endo-1 3-beta-glucosidase	d	6.4	−1.0	0.07
Solyc02g083760.1.1	sr	Thaumatin-like protein	u	6.7	0.6	0.10
Solyc04g080260.1.1	sr	Glucan endo-1 3-beta-glucosidase	u	9.7	0.2	0.05
Solyc06g072230.1.1	sr	Kunitz trypsin inhibitor	u	9.2	0.4	0.06
**Six6**						
Solyc08g005800.1.1	cw	Pectinacetylesterase like protein	d	8.7	−0.3	0.07
Solyc08g066810.1.1	cw	Glycosyl hydrolase family	d	7.5	−0.9	0.07
Solyc04g009630.1.1	cw	Alpha-glucosidase	u	7.9	0.7	0.09
Solyc11g005770.1.1	cw	Pectinesterase family protein	u	8.8	0.2	0.07
Solyc03g031800.1.1	cw	Xyloglucan endotransglucosylase/hydrolase	u	8.0	1.2	0.06
Solyc07g017600.1.1	cw	Pectinesterase	u	6.7	0.7	0.09
Solyc04g076190.1.1	d	Aspartic proteinase nepenthesin	d	8.6	−0.4	0.08
Solyc10g081510.1.1	m	5-methyltetrahydropteroyltriglutamate-homocysteine methyltransferase	d	9.1	−0.3	0.05
Solyc03g113030.1.1	m	Aldose 1-epimerase-like protein	u	7.4	0.7	0.09
Solyc06g082250.1.1	m	Laccase-13	u	6.8	1.2	0.06
Solyc07g007670.1.1	m	Purple acid phosphatase 3	u	7.8	1.2	0.06
Solyc01g112080.1.1	na	LysM-domain GPI-anchored protein	d	7.2	−0.9	0.09
Solyc10g074820.1.1	na	Unknown Protein	d	7.7	−0.3	0.06
Solyc10g081120.1.1	na	Alpha-L-arabinofuranosidase	d	9.6	−0.2	0.06
Solyc11g040330.1.1	na	Endo-1 4-beta-xylanase	d	6.4	−1.3	0.07
Solyc02g024050.1.1	o	Ferredoxin–NADP reductase	u	6.4	0.8	0.09
Solyc11g066620.1.1	p	Aspartyl protease family prote	d	7.2	−0.8	0.06
Solyc12g099160.1.1	p	Serine carboxypeptidase	d	8.5	−0.3	0.02
Solyc06g083030.1.1	p	Serine carboxypeptidase	u	7.9	1.0	0.10
Solyc07g041970.1.1	p	Subtilisin-like protease	u	7.2	1.6	0.01
Solyc02g077040.1.1	p	Cathepsin B-like cysteine proteinase	u	8.3	0.8	0.09
Solyc05g054710.1.1	p	Beta-hexosaminidase b	u	7.1	1.0	0.04
Solyc11g008810.1.1	p	Beta-hexosaminidase b	u	7.6	1.6	0.04
Solyc10g084320.1.1	p	Subtilisin-like protease	u	7.5	1.4	0.05
Solyc12g087940.1.1	p	Aspartic proteinase nepenthesin-1	u	6.7	1.1	0.07
Solyc12g088760.1.1	p	Subtilisin-like protease	u	9.3	0.5	0.05
Solyc05g052280.1.1	po	Peroxidase	d	8.5	−0.3	0.09
Solyc09g007520.1.1	po	Peroxidase	d	8.0	−0.4	0.07
Solyc01g105070.1.1	po	Peroxidase	d	9.2	−0.4	0.07
Solyc06g076630.1.1	po	Peroxidase	d	8.7	−0.2	0.07
Solyc12g013900.1.1	r	CT099	d	8.8	−0.3	0.09
Solyc02g094050.1.1	r	Blue copper protein	u	8.4	1.3	0.06
Solyc07g064240.1.1	r	CT099	u	7.1	1.5	0.02
Solyc12g094460.1.1	r	Laccase-2	u	7.5	1.3	0.06
Solyc01g108840.1.1	s	Receptor-like kinase	d	7.8	−0.4	0.05
Solyc02g093210.1.1	s	MAPprotein kinase-like protein	d	7.9	−0.4	0.07
Solyc07g055690.1.1	s	S-locus-specific glycoprotein	u	7.5	1.9	0.01
Solyc04g072000.1.1	sr	Chitinase	d	9.1	−0.3	0.03
Solyc06g072220.1.1	sr	Kunitz trypsin inhibitor	d	9.1	−0.2	0.07
Solyc06g072230.1.1	sr	Kunitz trypsin inhibitor	d	7.8	−0.5	0.05
Solyc10g074440.1.1	sr	Endochitinase	d	8.9	−0.4	0.08
Solyc10g079860.1.1	sr	Beta-1 3-glucanase	d	9.5	−0.6	0.02
Solyc09g007010.1.1	sr	Pathogenesis related protein PR-1	u	7.5	1.9	0.06
Solyc12g019890.1.1	sr	Glucan endo-1 3-beta-glucosidase	u	7.6	1.0	0.08

u, increased abundance; d, decreased abundance, Log10 Label Free Quantification (LFQ) values compared to Fol Wildtype (Fol007) and adjusted P-values (adjPval) are depicted. sr, stress responses; s, signaling; r, redox; po, peroxidases; p, protein modification/degradation; o, others; na, not assigned; m, metabolism; d, DNA/RNA related; cw, cell wall.

In the xylem sap proteome of *SIX5* knockout strain-treated plants 18 unique DAPs were identified (Figure 6A). When not only the absolute change in protein abundance was taken into account, as compared to Fol wild-type infected plants, but also the direction, in- or decrease, 6 additional ΔSIX5-specific DAPs were found: an unknown Protein (Solyc10g074820.1.1), a Peroxidase (Solyc09g007520.1.1), a Kunitz trypsin inhibitor (Solyc06g072230.1.1), a Glucan endo-1 3-beta-glucosidase (PR-2), (Solyc04g080260.1.1), a Receptor-like kinase (Solyc01g108840.1.1) and a Serine carboxypeptidase K10B2.2 (Solyc12g099160.1.1) (Table 2). The category “metabolism” is overrepresented as compared with the ΔAVR2-specific DAPs as 39% of all ΔSIX5-specific DAPs belong to this category (Figure 6B). No proteins from the category “cell wall” were found. Generally, more DAPs were increased (17) than decreased (7) in this xylem sap proteome. Remarkably, the enzymes phosphoglucomutase (Solyc04g045340.1.1) and Fructose-1-6-bisphosphatase (Solyc05g052600.1.1) were identified in the latter group of DAPs, which have catalytic functions in glycogenesis.

**Table 2:** **List of effector-common DAPs and their functional annotation**.

**ID**	**GO**	**Functional description**	**u(p)/d(own)**
Solyc07g006860.1.1	cw	Xyloglucan endotransglucosylase/hydrolase 3	u
Solyc06g062380.1.1	m	Acid phosphatase	u
Solyc12g009800.1.1	m	Purple acid phosphatase 3	u
Solyc06g007170.1.1	na	Protein of unknown function	u
Solyc01g080010.1.1	p	Xylanase inhibitor	d
Solyc08g079870.1.1	p	Subtilisin-like protease	d
Solyc07g041900.1.1	p	Cathepsin L-like cysteine proteinase	d
Solyc12g010040.1.1	p	Leucyl aminopeptidase	d
Solyc04g071890.1.1	po	Peroxidase 4	d
Solyc06g050440.1.1	po	Peroxidase	u
Solyc09g009390.1.1	r	Monodehydroascorbate reductase (NADH)-like protein	d
Solyc01g104400.1.1	r	Blue copper protein	u
Solyc10g085670.1.1	s	LRR receptor-like serine/threonine-protein kinase FEI 1	d/u
Solyc02g093310.1.1	s	MAP-like protein kinase	u
Solyc03g098670.1.1	sr	Kunitz trypsin inhibitor	d
Solyc04g016470.1.1	sr	Beta-1 3-glucanase	d
Solyc02g082920.1.1	sr	Endochitinase (Chitinase)	d
Solyc05g050130.1.1	sr	Acidic chitinase	d
Solyc00g174340.1.1	sr	Pathogenesis-related protein 1b	d
Solyc10g052880.1.1	sr	Leucine-rich repeat family protein	u
Solyc08g080620.1.1	sr	Osmotin-like protein (Fragment)	u
Solyc05g025500.1.1	sr	Glucan endo-1 3-beta-glucosidase 6	u
Solyc01g097270.1.1	sr	Chitinase	u/d
Solyc08g080640.1.1	sr	Osmotin-like protein	u

u, increased abundance; d, decreased abundance compared to xylem sap infected with Fol-wildtype; sr, stress responses; s, signaling; r, redox; po, peroxidases; p, protein modification/degradation; o, others; na, not assigned; m, metabolism; d, DNA/RNA related; cw, cell wall.

The 29 specific DAPs, and the 15 DAPs (Solyc12g099160.1.1, Solyc11g040330.1.1, Solyc10g074820.1.1, Solyc09g007520.1.1, Solyc06g072230.1.1, Solyc01g108840.1.1, Solyc09g007010.1.1, Solyc08g066810.1.1, Solyc06g072220.1.1, Solyc05g054710.1.1, Solyc05g052280.1.1, Solyc04g072000.1.1, Solyc02g077040.1.1, Solyc02g024050.1.1, and Solyc01g105070.1.1) whose abundance was either specifically increased or decreased in the xylem sap of Fol *SIX6* knockout compared to other effector knockout strains inoculated tomato plants (Table 2, Figure 6A) distribute over all GO categories except “others.” Relatively more DAPs belonging to the categories “protein modification and degradation” and “redox” were identified than in Fol007 vs. mock (Figure 6B) and relatively fewer proteins from “stress responses,” “peroxidases,” and “cell wall.” All peroxidases were decreased in abundance while the abundance of protein-degrading enzymes was mostly increased (Table 2).

In summary, knockout of a single effector gene in Fol specifically affects the composition of the xylem sap proteome of infected tomato plants. All effector knockout strains that were compromised in virulence affected the abundance of a core set of 24 xylem sap proteins. In addition, each effector knockout strain had a unique effect as shown by their specific GO fingerprint profile affecting distinctive DAPs. The *SIX2* knockout did not affect the xylem sap proteome in comparison to the wild-type. This is in line with the observation that disease development of tomato plants infected with the *SIX2* knockout strain is identical to that of plants infected with the wild-type strain.

## Discussion

### Changes in the xylem sap proteome upon fol infection

The tomato xylem sap proteome changes dramatically upon infection with Fol. Using a quantitative MS approach, 388 plant and 43 fungal proteins were identified when the data from both experiments were merged. When focusing on Experiment 1, 12 new tomato proteins were found while 45 proteins were no longer detected in the xylem sap proteome as compared to mock-infected plants. Gene ontology analysis revealed that mostly proteins from the categories “stress responses” (mostly PR proteins), “signaling” (many LRR class receptor like kinases), “protein modifications” (many peptidases) and “not assigned” were no longer detectable. This suggests that either expression of the encoding genes is reduced or that protein turnover or location is altered resulting in reduced abundances.

Of all proteins identified in both experiments 97% showed changes in abundance following infection. Xylem sap proteins are produced in the cells adjacent to the root xylem and subsequently secreted into the xylem sap (Kehr et al., 2005). Upon Fol infection, expression of xylem sap protein-encoding genes is likely to differ between colonized and uninfected tissues. Since the xylem sap proteome is produced by a combination of infected and non-infected root and xylem tissue, it is surprising that a relatively large number of proteins (45) disappeared from the proteome upon infection. This suggests a systemic signal originating from infected tissues that affects gene expression in healthy tissues. The nature of this signal is unknown, but could be one of the known root generated systemic signals (Fu and Dong, 2013).

In previous studies employing 1-D and 2-D gel electrophoresis, thirteen proteins, including PR-1, two PR-2 isoforms, PR-3, PR-5, and peroxidases, were found to accumulate in tomato xylem sap following inoculation with Fol (Rep et al., 2002; Houterman et al., 2007) whereas abundance of the Lipid Transfer Like protein (LTP) XSP10 was decreased (Rep et al., 2002, 2003). Silencing *XSP10* showed that the encoded protein is required for disease symptom development (Krasikov et al., 2011). Since expression of the *XSP10* gene is constitutive during infection it was suggested that either synthesis or secretion of XSP10 is suppressed or that XSP10 is turned over during infection (Rep et al., 2003). In this study a clear reduction in the abundance of XSP10 was observed in Experiment 1, but not in Experiment 2. Since Experiments 1 and 2 were similar regarding both biological and data-quality, this difference is noteworthy. Sampling of both experiments was performed at similar time points after infection. However, in the second experiment plants clearly showed less disease symptoms. This lower disease severity corresponds to the lower number of fungal proteins identified in the latter experiment (19 vs. 43), suggesting a less successful Fol infection (Figure 5A). Plant stage, fitness and the nutritional status as well as environmental conditions are known to determine the speed and severity of disease development (Yadeta and Thomma, 2013). Likely, those factors are affected by the season, explaining the quantitative difference in disease development and severity even though the experiments were conducted in climatized greenhouse compartments. A reduced severity of fungal infection may have caused the less effective reduction of XSP10 abundance in Experiment 2. However, because LFQ values between Experiments 1 and 2 were not directly comparable, we could not estimate whether the absolute abundance of XSP10 was indeed higher in Experiment 2 than in Experiment 1.

### One fourth of all xylem sap proteins does not carry a classical signal peptide

As xylem vessels consist of dead cells, xylem sap proteins originate from neighboring living cells. The vast majority of extracellular proteins are secreted through the endoplasmic reticulum—Golgi pathway (Kehr et al., 2005). Secretion of such proteins is mediated by an amino-terminal signal peptide and the majority (283) of the identified xylem sap proteins are indeed predicted to contain such a signal peptide. Of the 105 proteins that did not have a predicted signal peptide, 77 were suggested by SecretomeP to use the non-classical secretory pathway (N score > 0.5) (“unconventional secreted proteins”). The 28 remaining xylem sap proteins contained neither a signal peptide nor were predicted to be unconventionally secreted. Two of these, a beta-1,3-glucanase (PR-2) (Solyc02g086700.2.1) and a 5-methyltetrahydropteroyltriglutamate-homocysteine methyltransferase (Solyc10g081510.1.1), were highly abundant in both data sets having LFQ values larger than nine. Similarly, in the xylem sap of *Brassica oleracea* a putative intracellular methionine synthase was identified in relatively high amounts next to other less abundant intracellular proteins (Ligat et al., 2011). The overall low number of putative intracellular proteins retrieved in the xylem sap in our experiments makes it unlikely that these are contaminants originating from tissues other than xylem vessels. Possibly, these 28 proteins originated from developing xylem precursor cells that are released after cell death to form tracheary elements (Dafoe and Constabel, 2009; Yadeta and Thomma, 2013). Alternatively, annotation of some of these proteins could be incorrect or incomplete, resulting in a mis-annotation of the N-terminus (i.e., missing the signal peptide).

It has been proposed that non-classical protein secretion is preferably activated upon biotic and abiotic stresses (Agrawal et al., 2010). If so, one expects enrichment of proteins lacking a signal peptide in the proteome of Fol-infected plants as compared to the mock. To see if this was the case we looked at the abundance of the 77 xylem sap proteins without a predicted signal peptide before and after infection. Of the 12 plant proteins unique for the “infected” proteome only five are predicted to have a signal peptide—the other eleven do not. In the control proteome the majority (35) of the 45 unique proteins were predicted to contain a signal peptide. This appears to support the hypothesis of increased non-classical secretion upon infection. Within the population of DAPs present in both the “mock-” and “Fol-treated” proteome, no clear over-representation could be detected of proteins lacking a signal peptide, regardless of whether their abundance increased or decreased.

**Figure 7:**
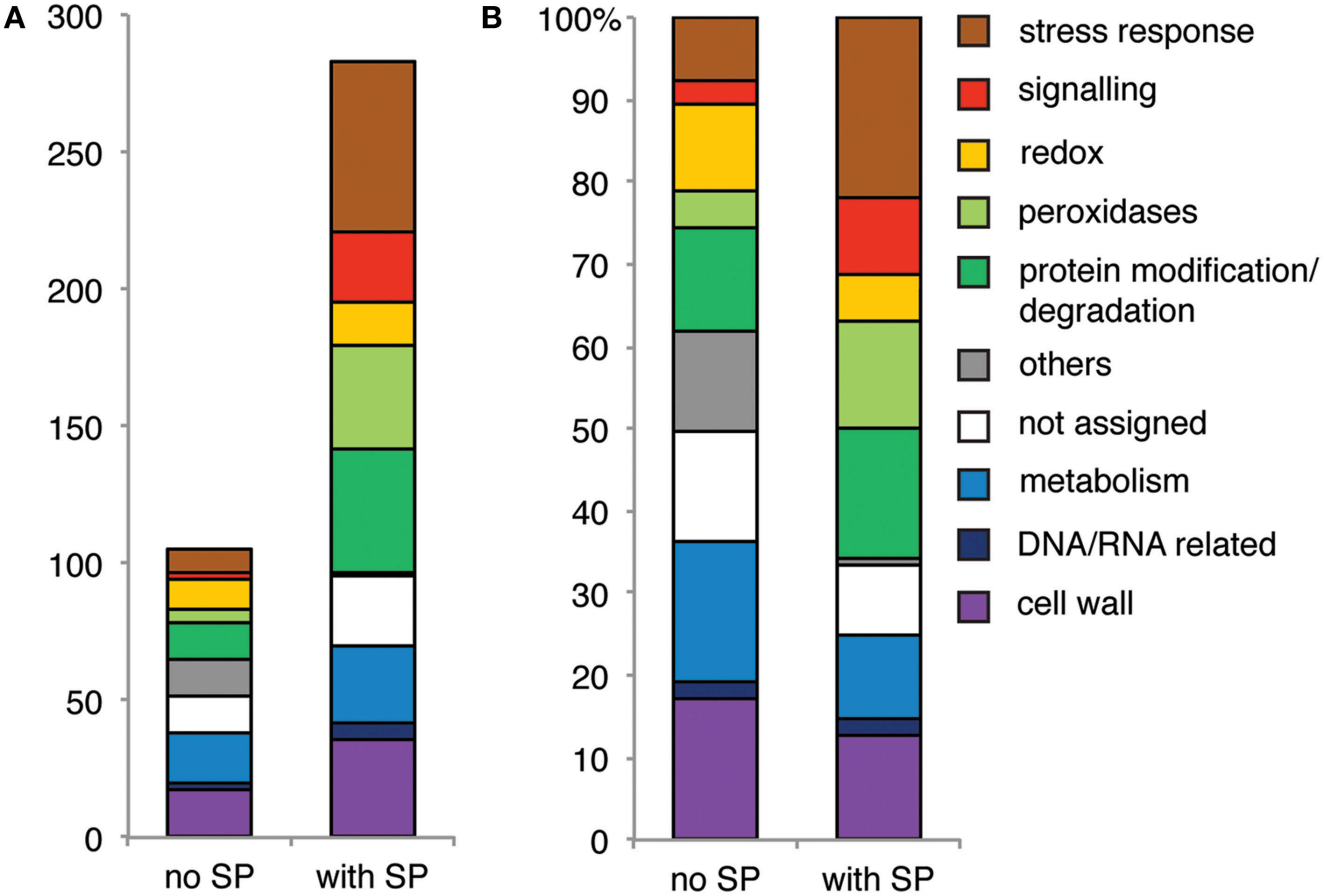
**Proteins with functions in stress responses and signaling and peroxidases generally carry a predicted signal peptide (SP)**. Bar chart showing **(A)** the number and **(B)** the percentage of all proteins identified in data set 1 and 2 per GO category. All identified proteins were separated into two classes (no SP, with SP).

Regarding the total protein dataset from both experiments, the majority of proteins related to “stress responses” had a signal peptide (Figure 7). The same was found for “peroxidases,” “signaling related proteins” and proteins associated to protein modification and degradation. Relatively speaking, proteins with a function in the “redox system,” in “metabolism” or in “cell wall” were more likely to be secreted via an alternative route. Apparently, non-classical-secretion preferably occurs for specific functional categories of xylem sap proteins, but the relationship between infection and their route of secretion needs to be clarified further.

### The absence of single effectors affects abundance of specific xylem sap proteins

Four out of five *SIX* gene knockouts had both shared and specific effects on the xylem sap proteome composition. These four *SIX* genes encode virulence factors (Avr3, Avr2, Six5, Six6) (Rep et al., 2005; Houterman et al., 2009; Gawehns et al., 2014). Avr2 and Six5 interact in a Yeast-Two-Hybrid and *in planta* and both are required to induce full resistance in *I-2* carrying plants (Ma et al., 2015). Here we observed a significant higher overlap between the DAPs of the *SIX5* and *AVR2* knockout than between other knockouts. This finding is in support of a functional overlap of these effectors, although they might also have specific activities.

The *SIX2* knockout was slightly compromised in virulence on seedlings, but its disease symptoms in 4-week-old plants were indistinguishable from the wild-type. Correspondingly, its xylem sap proteome was also identical as wild-type, which shows the robustness and reproducibility of the quantification method.

Characterization of effector action by analyzing the xylem sap proteome is a novel strategy to study microbial virulence. The most similar plant study that we found was one where a *Pseudomonas syringae* hrpA- strain, which is unable to secrete any type III secreted effectors (TTEs), was added to an *A. thaliana* cell suspension (Kaffarnik et al., 2009). In that study, among others, accumulation of a SOD was suppressed while an enolase was induced by the TTEs. Notably, abundance of a SOD was also significantly decreased in tomato xylem sap upon inoculation with the *AVR3* knockout strain. Hence, the presence of Avr3 apparently enhances SOD abundance. As a scavenger of reactive oxygen species (ROS) SOD functions in abiotic stress tolerance and its reduced abundance might be due to the reduced pathogenicity of the *AVR3* knockout strain.

Upon inoculation with the *AVR2* knockout strain the most notable increase in abundance was observed among cell wall degrading enzymes, specifically galactosidases, which degrade cell walls through galactomannan mobilization, glucosidases, which can hydrolyze xyloglucan but also salicylic acid or abscisic acid, glucosides and endo 1,4 xylanases that convert xylan into xylanose. Also a decrease was found for a TCTP, whose expression levels normally increase in response to abiotic stresses and upon pathogen infection (Berkowitz et al., 2008). In conclusion, Avr2 appears to suppress abundance of cell-wall degrading proteins while it triggers abundance of a TCTP.

The abundance of an enolase was decreased specifically upon inoculation with the *SIX5* knockout strain compared to the wild-type. Hence, Six5 may trigger enolase abundance, possibly in a manner comparable to the TTSs of *P. syringae.* Upon infection with both the *AVR2* and *SIX5* knockout strains, abundance of a thaumatin-like protein (PR-5) was higher than with WT infection. Furthermore, abundance of two other PR-5 isoforms was higher upon inoculation with both knockouts than upon infection with the wild-type fungus. Hence, the presence of Avr2 and Six5 together appear to have an inhibitory effect on the abundance of PR-5, a protein that has been proposed to display antifungal activities against i.a. *F. oxysporum* (Hu and Reddy, 1997).

Deletion of *SIX6* has the strongest effect on specific DAPs, altering abundance of 44 proteins; 22 proteins increased in abundance and 22 decreased. The largest functional groups of proteins affected are proteases and cell wall degrading enzymes, among which also proteins involved in pectin degradation. Notably, abundance of four peroxidases was reduced. Generally, peroxidases catalyze the oxidation of different substrates using H_2_O_2_ under the production of ROS. This process is active in different physiological processes like phenol oxidation and lignification, which play a role in the defense against pathogens and in cell wall formation (Passardi et al., 2005).

The altered virulence of an effector knockout strain corresponds to a unique “fingerprint” of the xylem sap proteome. We consider two scenarios to explain this observation: (I) the effector indirectly mediates abundance of a set of host proteins by affecting specific signaling pathways that control their expression or (II) the absence of an effector directly changes the xylem sap protein content, for instance by interacting with specific proteins, affecting their turnover or their mobility in the sap. The common effect on the xylem sap proteome composition induced by all effector knockouts except *SIX2* complies with the first scenario and is related to the degree of virulence of the strains. The affected group set was dominated by proteins from the group “stress responses,” including pathogenesis related (PR) proteins like PR-1, β-1,3-glucanases (PR-2), several chitinases (PR-3) and PR-5 (Table 2). This suggests a shared signaling pathway, which is manipulated only upon infection with a fully virulent pathogen, as the abundance of the proteins controlled by this pathway was altered upon infection of a knockout strain. The effector-specific proteome changes could fit either scenario. Future studies, aimed at revealing the identity of the signaling components targeted by the fungal effector proteins, are required to resolve this issue.

In summary, our study implies that the defense against a vascular pathogen like Fol is mostly based on changes in cell wall remodeling proteins and secretion of proteins with proposed antifungal activity. Individual Six proteins might play exclusive roles to overcome some of those physiological adaptations to allow the fungus to infect and spread.

## Author contributions

FG, LM, and PH collected the xylemsap and prepared the samples for MS analysis. SB performed the LC MS/MS measurements and FG, OB, and SB analyzed the data. BC provided intellectual input and critically evaluated the manuscript. FG, MR, and FT designed the experiments and FG and FT wrote the manuscript. All authors provided intellectual input and approved the manuscript and are accountable for accuracy and integrity of this study.

### Conflict of interest statement

The authors declare that the research was conducted in the absence of any commercial or financial relationships that could be construed as a potential conflict of interest.

## Supplementary material

Figure S1 **Pipeline for the identification and functional annotation of Differential Accumulated xylem sap Proteins (DAPs)**. Upon inoculation of tomato with different Fol strains the xylem sap was harvested after appearance of the first disease symptoms. The concentrated proteins were shortly run in an SDS-polyacrylamide gel electrophoresis (PAGE), protein-containing bands were cut out of the gel and used for in gel tryptic digestion. The obtained peptides were identified and quantified using nanoLC-MS/MS coupled to a bioinformatics pipeline (MaxQuant). The data were filtered on reliability, normalized and a mixed linear model was fitted. Differences of the xylem sap proteome between different treatments were tested for permutated significance resulting in the identification of DAPs, which were subsequently functionally annotated.

Figure S2 **The protein abundance distributions between replicates and treatments become more comparable after median normalization**. Boxplots showing the protein abundance distributions based on the LFQ values for Experiment 1 (top left) and experiment 2 (top right) and after median normalization on the LFQ values for Experiment 1 (bottom left) and Experiment 2 (bottom right).

Figure S3 **Differences between Fol- and mock-treated plants are similar for both experiments**. PCA plot based on the combined median normalized log10 data of the Fol- or mock-treated plants from both data sets. PC1 is plotted on the x-axis, PC2 is plotted on the y-axis.

Figure S4 **The data sets of Experiments 1 and 2 correlate**. Scatter plot generated from the FC (log10 Fold Change) values obtained in Experiments 1 and 2. The data points align on a line through 0 by trend.

Supplemental Table 1 **Overview of protein annotation information (columns A-G), SignalP analysis (column H), group averaged normalized log10 LFQ values from the ANOVA fit (columns I-Q), “Present,” “Marginal” and “Absent” calls (columns R-Z), normalized log10 LFQ ratio data (columns AA-AG), FDR adjusted p-values (columns AH-AN), DAPs increase or decrease (columns AO-AU), observed *F*-values from the F1 test (columns AV-BB), processed Max Quant log10 LFQ data (columns BC-CL)**.

Supplemental Table 2 **List of gene ontology terms categories grouped by the corresponding bin-codes**.

Supplemental Table 3 **Identified proteins and log10 LFQ values for dataset 1**. Table lists, identity (id), protein identity (Protein IDs and Majority Protein IDs), Peptide Counts (unique), Protein Descriptions, Proteins, Peptides, Unique Peptides and peptides identified in the different replica's. The samples are labeled as follows: “non-infected” for mock, “d65” for the Six6 knockout, “07” for Fol 007 wild-type, “dAvr2” for the Avr2 knockout and “d65” for the Six5 knockout. Sequence Coverage [%] and Mol. Weight [kDa] are given for all samples. Log10 LFQ values and MS/MS counts for the samples are provided including alternative identification methods: Only identified by site or Reverse. Contaminants are marked with a +.

Supplemental Table 4 **Identified proteins and log10 LFQ values for dataset 2**. Table lists, identity (id), protein identity (Protein IDs and Majority Protein IDs), Peptide Counts (unique), Protein Descriptions, Proteins, Peptides, Unique Peptides and peptides identified in the different replica's. The samples are labeled as follows: “d007” for Fol 007 wild-type, “Six1” for the Avr3 knockout, “Six2” for the Six2 knockout and “Six6” for the Six6 knockout. Sequence Coverage [%] and Mol. Weight [kDa] are given for all samples. LFQ values and MS/MS counts for the samples are provided including alternative identification methods: Only identified by site or Reverse. Contaminants are marked with a +.

Supplemental Table 5 **Differentially identified proteins in the xylem sap of Fol Wild-type (Fol007) and mock-inoculated tomato plants**. Protein identity (ID), functional description and Log10 LFQ values of Fol007, Mock and the contrasts are depicted together with adjusted *P*-values (adjPval).
