# Supplementary figures and images for: The effector repertoire of Fusarium oxysporum determines the tomato xylem proteome composition following infection
Source: Front Plant Sci. 2015 Nov 4;6:967. doi: 10.3389/fpls.2015.00967 (PMC4631825; doi:10.3389/fpls.2015.00967)

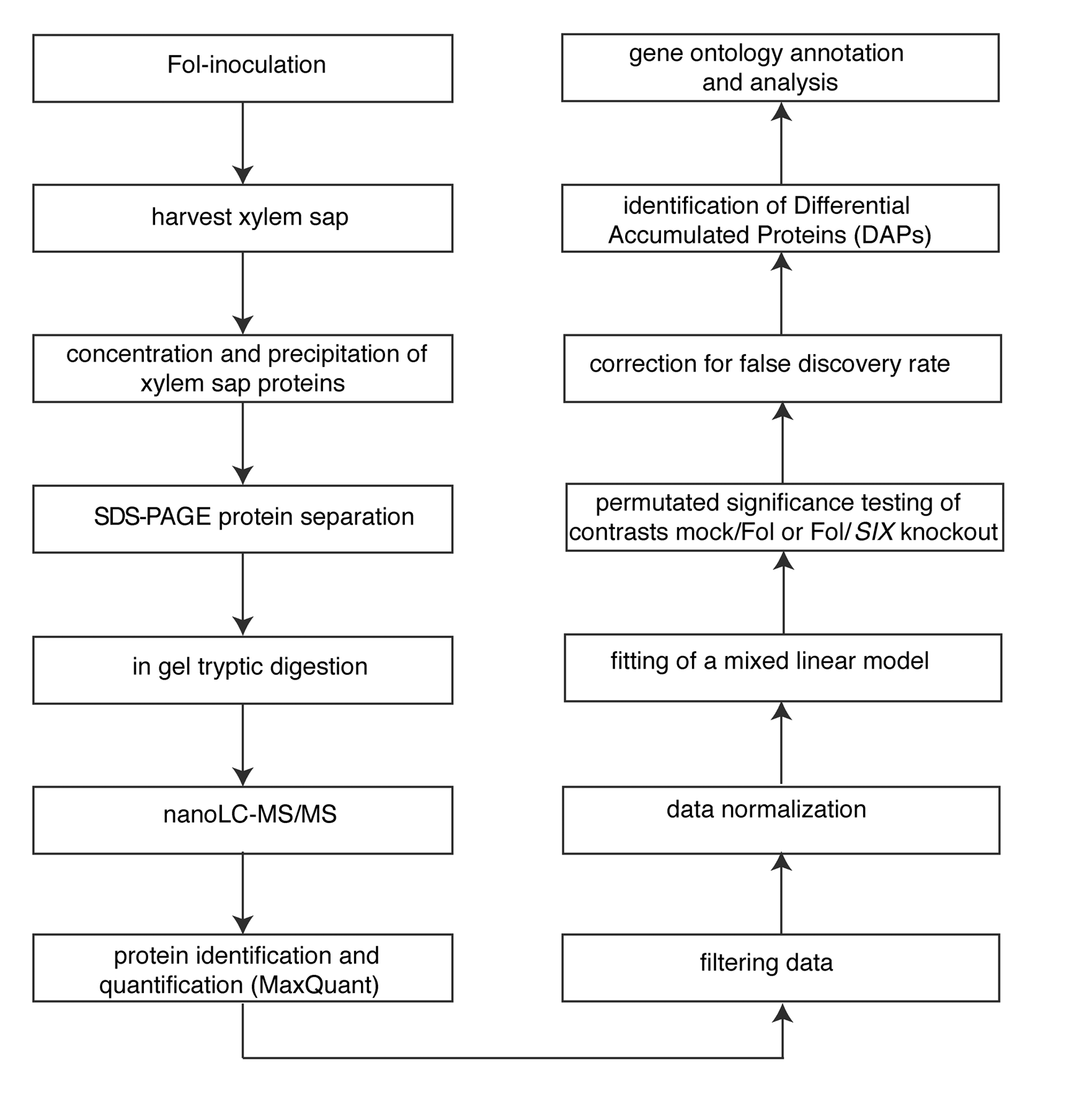

Supplement: Figure S1 — Pipeline for the identification and functional annotation of Differential Accumulated xylem sap Proteins (DAPs). Upon inoculation of tomato with different Fol strains the xylem sap was harvested after appearance of the first disease symptoms. The concentrated proteins were shortly run in an SDS-polyacrylamide gel electrophoresis (PAGE), protein-containing bands were cut out of the gel and used for in gel tryptic digestion. The obtained peptides were identified and quantified using nanoLC-MS/MS coupled to a bioinformatics pipeline (MaxQuant). The data were filtered on reliability, normalized and a mixed linear model was fitted. Differences of the xylem sap proteome between different treatments were tested for permutated significance resulting in the identification of DAPs, which were subsequently functionally annotated. [file Image1.TIFF]

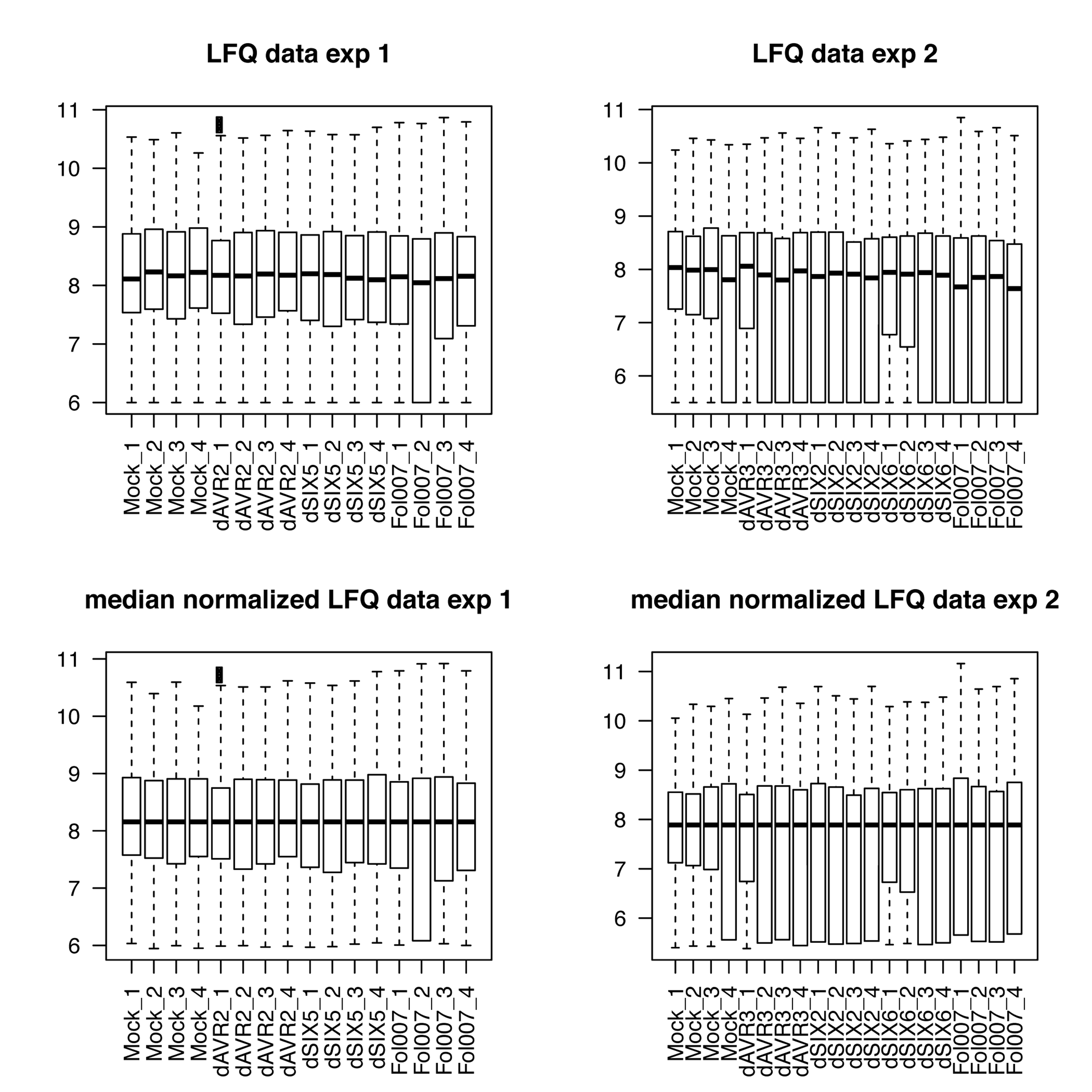

Supplement: Figure S2 — The protein abundance distributions between replicates and treatments become more comparable after median normalization. Boxplots showing the protein abundance distributions based on the LFQ values for Experiment 1 (top left) and experiment 2 (top right) and after median normalization on the LFQ values for Experiment 1 (bottom left) and Experiment 2 (bottom right). [file Image2.TIFF]

**combined data Data set 1 and 2**

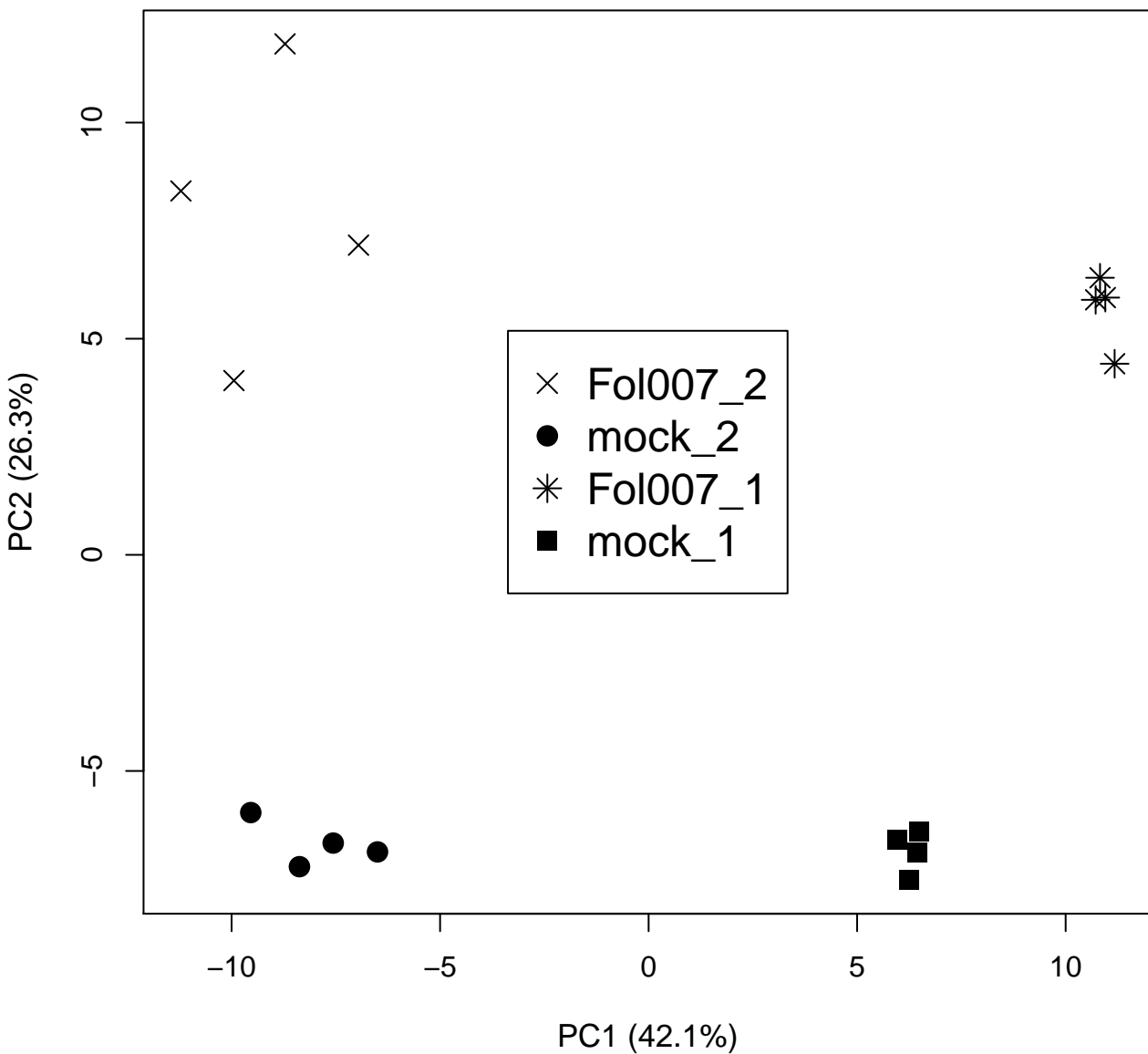

Supplement: Figure S3 — Differences between Fol- and mock-treated plants are similar for both experiments. PCA plot based on the combined median normalized log10 data of the Fol- or mock-treated plants from both data sets. PC1 is plotted on the x-axis, PC2 is plotted on the y-axis. [file Image3.PDF]

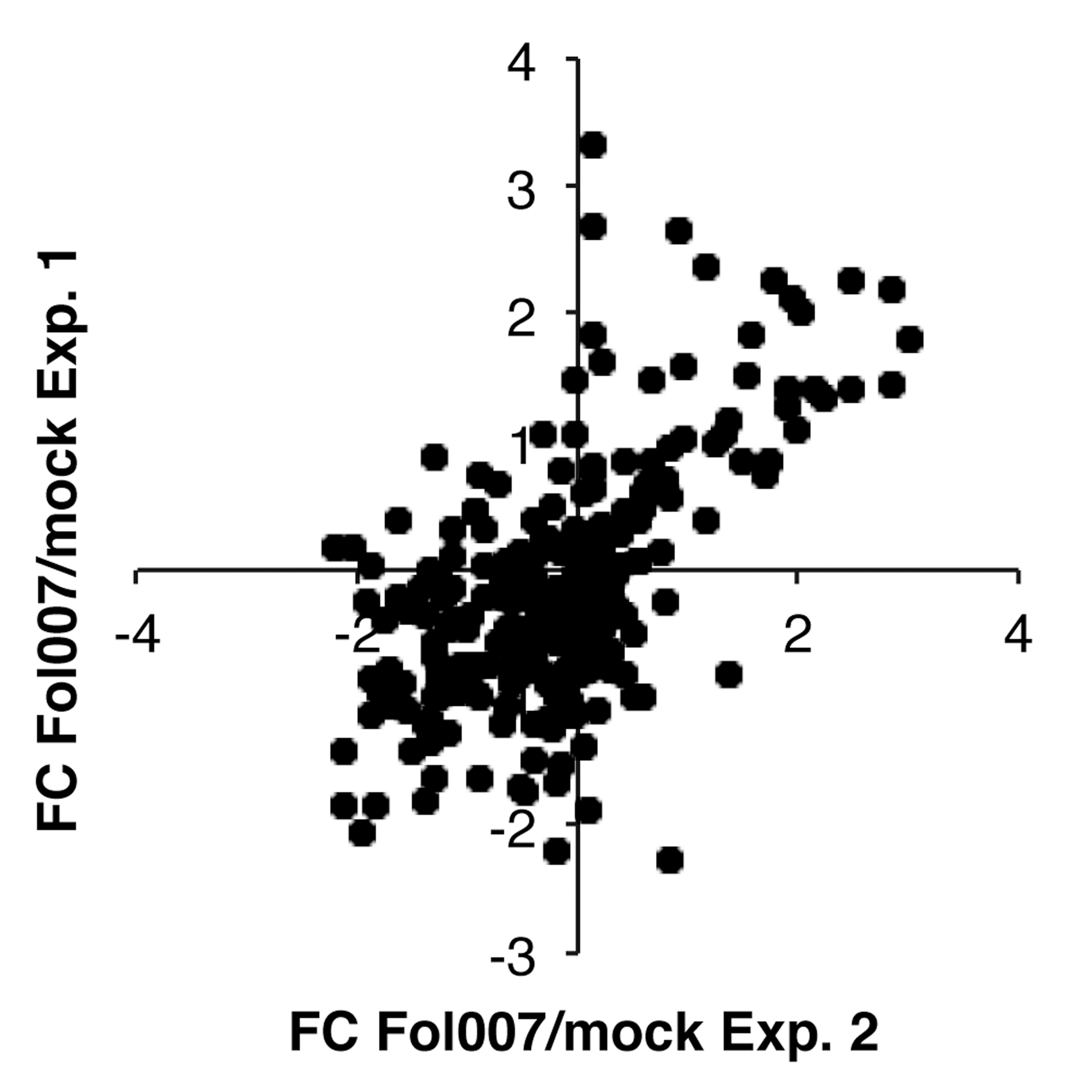

Supplement: Figure S4 — The data sets of Experiments 1 and 2 correlate. Scatter plot generated from the FC (log10 Fold Change) values obtained in Experiments 1 and 2. The data points align on a line through 0 by trend. [file Image4.TIFF]
