# Supplementary material for: The effector repertoire of Fusarium oxysporum determines the tomato xylem proteome composition following infection
Source: Front Plant Sci. 2015 Nov 4;6:967. doi: 10.3389/fpls.2015.00967 (PMC4631825; doi:10.3389/fpls.2015.00967)
Supplement: Supplemental Table 2 — List of gene ontology terms categories grouped by the corresponding bin-codes. [file Table2.DOCX]

**Supplemental Table 2:** List of gene ontology terms categories grouped by the corresponding bin-codes.

| **others** |
| --- |
| biodegradation of xenobiotics |
| cell cycle peptidylprolyl isomerase |
| development unspecified |
| transport metabolite transporters at the mitochondrial membrane |
| oxidative pentose phosphate cycle |
| photosynthesis lightreaction other electron carrier (ox/red) ferredoxin |
| photosynthesis calvin cycle |
| **cell wall** |
| cell wall proteins |
| cell wall degradation |
| cell wall modification |
| cell wall pectinesterases |
| misc. invertase/pectin methylesterase inhibitor family protein |
| misc. gluco-, galacto- and mannosidases alpha/beta-galactosidase |
| **metabolism** |
| lipid metabolism |
| misc. protease inhibitor/seed storage/lipid transfer protein (LTP) family protein |
| amino acid metabolism |
| secondary metabolism simple phenols |
| hormone metabolism auxin induced-regulated-responsive-activated |
| major CHO metabolism degradation sucrose |
| misc. nitrilases, nitrile lyases, berberine bridge enzymes, reticuline oxidases, troponine reductases |
| nucleotide metabolism degradation |
| nucleotide metabolism phosphotransfer and pyrophosphatases |
| minor CHO metabolism |
| glycolysis cytosolic branch |
| tricarboxylic acid cycle / org transformation |
| misc. acid and other phosphatases |
| **stress response** |
| stress biotic |
| stress biotic PR-proteins |
| misc. beta 1,3 glucan hydrolases |
| stress abiotic unspecified |
| misc. GDSL-motif lipase (biotic stress) |
| **redox** |
| redox thioredoxin |
| redox ascorbate and glutathione |
| redox dismutases and catalases |
| misc. plastocyanin-like |
| misc. oxidases - copper, flavone etc |
| misc. glutathione S transferases |
| **peroxidases** |
| misc. peroxidases |
| **DNA/RNA** |
| RNA processing ribonucleases |
| RNA regulation of transcription unclassified |
| DNA synthesis/chromatin structure |
| **protein** |
| protein targeting secretory pathway unspecified |
| protein degradation |
| protein glycosylation alpha-1,3/1,6-mannosyl-glycoprotein-beta-1,2-N-acetylglucosaminyltransferase(GnTI) |
| **signalling** |
| signalling receptor kinases |
| signalling calcium |
| signalling MAP kinases |
| signalling 14-3-3 proteins |
| PLP2/4 (lipid acyl hydrolase activity) derfence and signallng) |
| protein postranslational modification kinase receptor like cytoplasmatic kinase VII |
| **not assigned** |
| not assigned |
