# Supplementary material for: The effector repertoire of Fusarium oxysporum determines the tomato xylem proteome composition following infection
Source: Front Plant Sci. 2015 Nov 4;6:967. doi: 10.3389/fpls.2015.00967 (PMC4631825; doi:10.3389/fpls.2015.00967)
Supplement: Supplemental Table 5 — Differentially identified proteins in the xylem sap of Fol Wild-type (Fol007) and mock-inoculated tomato plants. Protein identity (ID), functional description and Log10 LFQ values of Fol007, Mock and the contrasts are depicted together with adjusted P-values (adjPval). [file Table5.DOCX]

| **ID** | **Functional Description** | **log10 LFQ Fol007** | **log10 LFQ mock** | **log10 FC Fol007 vs. mock** | **adjPval Fol007 vs. mock** |
| --- | --- | --- | --- | --- | --- |
| Solyc02g069670.1.1 | Alpha-glucosidase 1 | 6,4 | 6,6 | -0,2 | 0,08 |
| Solyc00g058890.1.1 | Unknown Protein | 6,0 | 6,9 | -0,8 | 0,02 |
| Solyc00g072400.1.1 | Peroxidase 1 | 6,4 | 7,9 | -1,5 | 0,00 |
| Solyc02g080220.1.1 | Pectinesterase | 7,2 | 7,1 | 0,1 | 0,12 |
| Solyc00g174340.1.1 | Pathogenesis-related protein 1b | 10,8 | 9,0 | 1,8 | 0,00 |
| Solyc01g006290.1.1 | Peroxidase | 8,6 | 8,2 | 0,4 | 0,00 |
| Solyc01g006300.1.1 | Peroxidase | 9,6 | 9,0 | 0,6 | 0,00 |
| Solyc01g006310.1.1 | Peroxidase | 8,8 | 8,5 | 0,3 | 0,05 |
| Solyc01g008620.1.1 | Beta-1 3-glucanase | 10,4 | 8,2 | 2,2 | 0,00 |
| Solyc01g009810.1.1 | LRR receptor-like serine/threonine-protein kinase FEI 1 | 9,0 | 7,7 | 1,3 | 0,00 |
| Solyc01g010710.1.1 | Serine carboxypeptidase 1 | 7,9 | 8,9 | -1,0 | 0,00 |
| Solyc03g083720.1.1 | Pectinesterase | 6,0 | 8,1 | -2,1 | 0,00 |
| Solyc01g059980.1.1 | Beta-glucanase | 9,4 | 8,4 | 1,0 | 0,00 |
| Solyc04g007150.1.1 | Alpha-glucosidase | 8,5 | 8,8 | -0,2 | 0,00 |
| Solyc01g067860.1.1 | Peroxidase 24 | 6,9 | 7,9 | -1,1 | 0,00 |
| Solyc01g068130.1.1 | Os05g0272200 protein | 8,5 | 9,3 | -0,8 | 0,00 |
| Solyc01g068380.1.1 | Purple acid phosphatase | 6,0 | 7,0 | -1,0 | 0,01 |
| Solyc01g079160.1.1 | GDSL esterase/lipase At1g09390 | 8,8 | 9,3 | -0,5 | 0,00 |
| Solyc01g079940.1.1 | Xylanase inhibitor | 6,6 | 6,7 | -0,1 | 0,10 |
| Solyc01g079950.1.1 | Xylanase inhibitor | 8,1 | 8,8 | -0,7 | 0,00 |
| Solyc01g080010.1.1 | Xylanase inhibitor | 9,1 | 7,6 | 1,5 | 0,00 |
| Solyc01g080790.1.1 | PBSP domain-containing protein | 7,3 | 8,0 | -0,7 | 0,02 |
| Solyc01g081610.1.1 | Beta-hexosaminidase 1 | 8,9 | 8,9 | -0,1 | 0,11 |
| Solyc01g087610.1.1 | Alpha-N-acetylglucosaminidase | 7,7 | 8,0 | -0,3 | 0,01 |
| Solyc04g007160.1.1 | Alpha-glucosidase | 6,0 | 6,6 | -0,6 | 0,04 |
| Solyc04g009630.1.1 | Alpha-glucosidase 1 | 7,6 | 7,6 | 0,0 | 0,14 |
| Solyc01g087850.1.1 | Subtilisin-like protease | 6,0 | 7,9 | -1,8 | 0,00 |
| Solyc01g088590.1.1 | Invertase inhibitor | 7,4 | 8,0 | -0,7 | 0,00 |
| Solyc04g077190.1.1 | Endo-1 4-beta-xylanase | 8,7 | 9,3 | -0,6 | 0,00 |
| Solyc01g090120.1.1 | Blue copper protein | 7,5 | 8,1 | -0,7 | 0,00 |
| Solyc01g091230.1.1 | Serine/threonine-protein kinase receptor | 6,4 | 7,1 | -0,7 | 0,00 |
| Solyc01g091530.1.1 | Fasciclin-like arabinogalactan protein 13 | 9,1 | 9,1 | 0,0 | 0,13 |
| Solyc01g096450.1.1 | Aspartic proteinase nepenthesin-1 | 9,7 | 10,2 | -0,5 | 0,00 |
| Solyc01g097240.1.1 | Pathogenesis-related protein 4B | 10,5 | 9,1 | 1,4 | 0,00 |
| Solyc01g097270.1.1 | Chitinase | 8,8 | 8,0 | 0,8 | 0,00 |
| Solyc05g049980.1.1 | Glycoside hydrolase family 28 protein/polygalacturonase family protein | 9,5 | 9,6 | -0,1 | 0,05 |
| Solyc07g048090.1.1 | Fasciclin-like arabinogalactan protein 2 | 8,3 | 8,5 | -0,2 | 0,00 |
| Solyc01g098780.1.1 | Signal peptidase complex subunit 3 | 6,0 | 7,2 | -1,2 | 0,01 |
| Solyc08g005800.1.1 | Pectinacetylesterase like protein | 9,3 | 9,1 | 0,3 | 0,01 |
| Solyc01g100090.1.1 | Wall-associated receptor kinase-like 20 | 8,6 | 10,1 | -1,4 | 0,00 |
| Solyc01g100380.1.1 | Calreticulin 2 calcium-binding protein | 8,4 | 8,1 | 0,4 | 0,00 |
| Solyc01g100930.1.1 | GDSL esterase/lipase At1g09390 | 6,0 | 7,2 | -1,2 | 0,02 |
| Solyc01g102330.1.1 | Acetyl xylan esterase A | 7,7 | 9,0 | -1,3 | 0,00 |
| Solyc01g102400.1.1 | Germin-like protein | 8,2 | 9,3 | -1,1 | 0,00 |
| Solyc01g104020.1.1 | Embryo-specific 3 | 6,0 | 7,8 | -1,8 | 0,00 |
| Solyc01g104400.1.1 | Blue copper protein | 7,5 | 7,7 | -0,2 | 0,08 |
| Solyc01g104950.1.1 | Alpha-L-arabinofuranosidase/beta-D-xylosidase | 9,3 | 9,2 | 0,1 | 0,05 |
| Solyc08g081480.1.1 | Polygalacturonase-like protein | 9,0 | 8,7 | 0,3 | 0,01 |
| Solyc01g106600.1.1 | Pathogenesis-related protein 1 | 7,1 | 6,8 | 0,2 | 0,11 |
| Solyc01g106620.1.1 | Pathogenesis-related protein 1a | 9,4 | 8,4 | 1,1 | 0,00 |
| Solyc01g107670.1.1 | Leucine-rich repeat receptor-like protein kinase | 6,4 | 8,3 | -1,9 | 0,00 |
| Solyc01g107790.1.1 | Leucine-rich repeat receptor-like protein kinase PEPR2 | 6,0 | 6,7 | -0,7 | 0,03 |
| Solyc01g108320.1.1 | Peroxidase | 7,0 | 7,6 | -0,6 | 0,04 |
| Solyc01g108840.1.1 | Receptor-like kinase | 8,0 | 8,4 | -0,4 | 0,00 |
| Solyc01g109570.1.1 | Glucan endo-1 3-beta-glucosidase 7 | 9,1 | 9,0 | 0,1 | 0,07 |
| Solyc09g075460.1.1 | Polygalacturonase-like protein-like | 8,4 | 8,6 | -0,2 | 0,02 |
| Solyc09g092160.1.1 | Beta-galactosidase | 7,4 | 8,6 | -1,2 | 0,00 |
| Solyc10g083810.1.1 | Pectinesterase | 6,0 | 6,6 | -0,5 | 0,04 |
| Solyc01g112000.1.1 | Expansin-like protein | 8,5 | 9,5 | -1,1 | 0,00 |
| Solyc01g112080.1.1 | LysM-domain GPI-anchored protein | 7,9 | 7,4 | 0,5 | 0,00 |
| Solyc02g022850.1.1 | FAD-binding domain-containing protein | 6,0 | 6,9 | -0,9 | 0,03 |
| Solyc11g005770.1.1 | Pectinesterase family protein | 8,8 | 8,9 | -0,1 | 0,10 |
| Solyc02g061770.1.1 | Endochitinase | 9,6 | 8,6 | 1,0 | 0,00 |
| Solyc02g064970.1.1 | Peroxidase | 9,0 | 9,4 | -0,4 | 0,02 |
| Solyc02g065470.1.1 | Pathogenesis-related protein | 8,8 | 8,5 | 0,3 | 0,01 |
| Solyc11g069270.1.1 | Beta-galactosidase | 9,6 | 10,1 | -0,5 | 0,00 |
| Solyc09g090990.1.1 | Major allergen Mal d 1 | 8,9 | 6,3 | 2,6 | 0,00 |
| Solyc02g070070.1.1 | FAD-binding domain-containing protein | 6,0 | 7,3 | -1,3 | 0,00 |
| Solyc02g076980.1.1 | Cathepsin B-like cysteine proteinase | 9,1 | 7,7 | 1,4 | 0,00 |
| Solyc09g091000.1.1 | Major allergen Mal d 1 | 9,6 | 6,5 | 3,0 | 0,00 |
| Solyc01g089970.1.1 | Nucleoside diphosphate kinase | 8,3 | 7,8 | 0,5 | 0,04 |
| Solyc02g079500.1.1 | Peroxidase | 9,7 | 9,1 | 0,6 | 0,00 |
| Solyc02g079510.1.1 | Peroxidase | 6,5 | 6,8 | -0,3 | 0,11 |
| Solyc01g106430.1.1 | Inorganic pyrophosphatase family protein | 7,1 | 6,0 | 1,1 | 0,00 |
| Solyc02g080630.1.1 | Lactoylglutathione lyase | 6,5 | 6,4 | 0,1 | 0,14 |
| Solyc02g082920.1.1 | Endochitinase | 10,4 | 9,5 | 0,8 | 0,00 |
| Solyc02g082930.1.1 | Endochitinase | 9,2 | 9,2 | 0,1 | 0,11 |
| Solyc02g083460.1.1 | Aspartyl protease family protein | 8,2 | 8,5 | -0,3 | 0,01 |
| Solyc02g083760.1.1 | Thaumatin-like protein | 6,0 | 7,7 | -1,6 | 0,00 |
| Solyc01g110450.1.1 | NADP dependent sorbitol 6-phosphate dehydrogenase | 8,0 | 6,5 | 1,5 | 0,00 |
| Solyc02g084780.1.1 | Peroxidase | 8,0 | 9,1 | -1,2 | 0,00 |
| Solyc02g084790.1.1 | Peroxidase | 7,4 | 8,5 | -1,1 | 0,00 |
| Solyc01g111210.1.1 | Glutamyl-tRNA | 6,8 | 7,8 | -1,0 | 0,01 |
| Solyc04g072280.1.1 | Laccase | 7,4 | 6,0 | 1,4 | 0,00 |
| Solyc02g087250.1.1 | Group II intron splicing factor CRS1-like | 7,1 | 7,7 | -0,5 | 0,04 |
| Solyc02g087520.1.1 | Thaumatin-like protein | 8,5 | 9,1 | -0,6 | 0,00 |
| Solyc04g081400.1.1 | Hexokinase 1 | 7,1 | 6,2 | 0,9 | 0,00 |
| Solyc02g088820.1.1 | Serine carboxypeptidase K10B2.2 | 8,3 | 7,3 | 1,1 | 0,00 |
| Solyc02g090450.1.1 | Peroxidase | 8,3 | 8,9 | -0,6 | 0,00 |
| Solyc02g090470.1.1 | Peroxidase | 9,0 | 9,5 | -0,5 | 0,00 |
| Solyc02g090490.1.1 | Patatin-like protein 3 | 7,7 | 8,5 | -0,8 | 0,00 |
| Solyc02g091680.1.1 | Alpha-L-arabinofuranosidase/beta-D-xylosidase | 7,0 | 7,5 | -0,5 | 0,05 |
| Solyc02g092580.1.1 | Peroxidase | 8,1 | 8,8 | -0,7 | 0,03 |
| Solyc02g092670.1.1 | Subtilisin-like protease | 10,0 | 10,0 | 0,0 | 0,12 |
| Solyc02g093210.1.1 | MAPprotein kinase-like protein | 8,6 | 8,6 | 0,0 | 0,14 |
| Solyc02g093310.1.1 | MAP-like protein kinase | 7,8 | 8,2 | -0,4 | 0,00 |
| Solyc02g094050.1.1 | Blue copper protein | 8,1 | 8,2 | -0,1 | 0,12 |
| Solyc02g094180.1.1 | Peroxidase 1 | 8,7 | 9,0 | -0,2 | 0,01 |
| Solyc03g006220.1.1 | Os06g0207500 protein | 8,3 | 9,2 | -0,9 | 0,00 |
| Solyc03g006700.1.1 | Peroxidase | 8,8 | 7,1 | 1,7 | 0,00 |
| Solyc03g019790.1.1 | Alpha-galactosidase | 8,9 | 9,5 | -0,7 | 0,00 |
| Solyc03g020040.1.1 | Proteinase inhibitor II | 7,5 | 8,8 | -1,3 | 0,00 |
| Solyc03g025380.1.1 | Peroxidase | 6,9 | 6,0 | 0,9 | 0,01 |
| Solyc03g031800.1.1 | Xyloglucan endotransglucosylase/hydrolase 1 | 8,0 | 8,3 | -0,3 | 0,00 |
| Solyc03g033620.1.1 | Serine carboxypeptidase S28 family protein | 8,3 | 8,5 | -0,3 | 0,05 |
| Solyc03g044100.1.1 | Peroxidase 5 | 9,5 | 10,0 | -0,5 | 0,00 |
| Solyc03g044150.1.1 | Subtilisin-like protease | 9,0 | 9,2 | -0,2 | 0,10 |
| Solyc03g079880.2.1 | Protease inhibitor/seed storage/lipid transfer protein family protein | 9,4 | 10,4 | -1,0 | 0,00 |
| Solyc03g080150.1.1 | Peroxidase 1 | 6,0 | 7,0 | -1,0 | 0,00 |
| Solyc05g014470.1.1 | Glyceraldehyde 3-phosphate dehydrogenase | 8,6 | 7,9 | 0,8 | 0,00 |
| Solyc03g083900.1.1 | Laccase-22 | 8,6 | 8,2 | 0,4 | 0,01 |
| Solyc03g093080.1.1 | Xyloglucan endotransglucosylase/hydrolase 9 | 8,6 | 9,3 | -0,7 | 0,00 |
| Solyc03g093360.1.1 | Wound/stress protein | 6,0 | 7,1 | -1,1 | 0,01 |
| Solyc03g098670.1.1 | Kunitz trypsin inhibitor | 8,8 | 6,6 | 2,3 | 0,00 |
| Solyc03g098730.1.1 | Kunitz trypsin inhibitor | 9,2 | 7,6 | 1,6 | 0,00 |
| Solyc03g098740.1.1 | Kunitz trypsin inhibitor | 9,0 | 7,9 | 1,1 | 0,00 |
| Solyc03g098780.1.1 | Kunitz-type protease inhibitor | 6,8 | 7,2 | -0,3 | 0,10 |
| Solyc03g111670.1.1 | Receptor-like protein kinase 1-like | 7,5 | 7,7 | -0,2 | 0,05 |
| Solyc03g113030.1.1 | Aldose 1-epimerase-like protein | 6,4 | 7,2 | -0,8 | 0,02 |
| Solyc03g115200.1.1 | Glucan endo-1 3-beta-glucosidase 1 | 8,5 | 7,8 | 0,7 | 0,04 |
| Solyc06g071920.1.1 | Glyceraldehyde-3-phosphate dehydrogenase | 8,0 | 6,5 | 1,5 | 0,00 |
| Solyc06g073190.1.1 | Fructokinase-like | 8,2 | 7,7 | 0,5 | 0,01 |
| Solyc06g073280.1.1 | LL-diaminopimelate aminotransferase | 6,9 | 6,0 | 0,9 | 0,01 |
| Solyc07g037960.1.1 | Ectonucleotide pyrophosphatase/phosphodiesterase 1 | 8,1 | 8,5 | -0,4 | 0,03 |
| Solyc07g048060.1.1 | Auxin-induced protein-like | 6,9 | 7,4 | -0,5 | 0,08 |
| Solyc04g007910.1.1 | Glucan endo-1 3-beta-glucosidase 3 | 7,4 | 7,8 | -0,4 | 0,09 |
| Solyc04g008730.1.1 | Alpha-galactosidase 1 | 7,4 | 8,0 | -0,6 | 0,04 |
| Solyc07g052350.1.1 | 3-isopropylmalate dehydratase large subunit | 6,9 | 6,0 | 0,9 | 0,01 |
| Solyc07g062650.1.1 | Malate dehydrogenase | 8,9 | 8,1 | 0,9 | 0,00 |
| Solyc08g041870.1.1 | Aspartate aminotransferase | 7,9 | 7,1 | 0,7 | 0,01 |
| Solyc04g015510.1.1 | Unknown Protein | 6,0 | 7,3 | -1,2 | 0,00 |
| Solyc04g016470.1.1 | Beta-1 3-glucanase | 9,7 | 7,7 | 2,0 | 0,00 |
| Solyc09g009020.1.1 | Enolase | 8,2 | 8,0 | 0,2 | 0,07 |
| Solyc04g054980.1.1 | Lipoxygenase homology domain-containing protein 1 | 6,0 | 7,7 | -1,7 | 0,00 |
| Solyc04g071890.1.1 | Peroxidase 4 | 9,8 | 8,0 | 1,8 | 0,00 |
| Solyc04g071900.1.1 | Peroxidase | 9,3 | 8,7 | 0,7 | 0,00 |
| Solyc04g072000.1.1 | Chitinase | 9,7 | 9,9 | -0,2 | 0,01 |
| Solyc09g090140.1.1 | Malate dehydrogenase | 8,7 | 7,4 | 1,3 | 0,00 |
| Solyc04g072860.1.1 | Beta-D-xylosidase | 8,3 | 8,3 | 0,0 | 0,15 |
| Solyc04g076190.1.1 | Aspartic proteinase nepenthesin-1 | 8,5 | 8,4 | 0,0 | 0,13 |
| Solyc04g076660.1.1 | Rhamnogalacturonate lyase | 6,0 | 6,8 | -0,8 | 0,03 |
| Solyc10g081510.1.1 | 5-methyltetrahydropteroyltriglutamate--homocysteine methyltransferase | 9,6 | 9,3 | 0,3 | 0,04 |
| Solyc04g077670.1.1 | Serine carboxypeptidase 1 | 6,0 | 6,6 | -0,6 | 0,01 |
| Solyc04g078110.1.1 | Subtilisin-like protease | 8,9 | 9,0 | -0,2 | 0,08 |
| Solyc04g078740.1.1 | Subtilisin-like protease | 9,2 | 9,4 | -0,3 | 0,05 |
| Solyc04g080260.1.1 | Glucan endo-1 3-beta-glucosidase 7 | 9,6 | 9,9 | -0,3 | 0,00 |
| Solyc01g099770.1.1 | Translationally-controlled tumor protein homolog | 8,2 | 8,0 | 0,2 | 0,05 |
| Solyc04g081550.1.1 | Thaumatin-like protein | 8,4 | 8,7 | -0,4 | 0,01 |
| Solyc04g081810.1.1 | Subtilisin-like protease | 9,8 | 10,3 | -0,5 | 0,00 |
| Solyc04g082140.1.1 | Laccase-22 | 8,7 | 9,4 | -0,7 | 0,00 |
| Solyc02g078650.1.1 | Polyphenol oxidase | 8,5 | 7,4 | 1,1 | 0,00 |
| Solyc05g007940.1.1 | Ribonuclease T2 | 8,2 | 8,8 | -0,6 | 0,00 |
| Solyc05g007950.1.1 | Ribonuclease T2 | 7,6 | 8,1 | -0,6 | 0,03 |
| Solyc04g045340.1.1 | Phosphoglucomutase | 7,3 | 6,5 | 0,9 | 0,02 |
| Solyc05g009430.1.1 | Endonuclease | 7,7 | 7,9 | -0,2 | 0,01 |
| Solyc05g009470.1.1 | Alpha-glucosidase | 7,8 | 8,6 | -0,8 | 0,02 |
| Solyc05g012260.1.1 | Purple acid phosphatase | 7,8 | 9,0 | -1,2 | 0,00 |
| Solyc05g013720.1.1 | Alpha-galactosidase | 7,8 | 8,3 | -0,5 | 0,02 |
| Solyc06g007170.1.1 | Os06g0207500 protein | 8,5 | 9,5 | -1,0 | 0,00 |
| Solyc05g015150.1.1 | LRR receptor-like serine/threonine-protein kinase FEI 1 | 6,9 | 7,5 | -0,6 | 0,03 |
| Solyc05g025500.1.1 | Glucan endo-1 3-beta-glucosidase 6 | 6,0 | 8,1 | -2,0 | 0,00 |
| Solyc05g046010.1.1 | Peroxidase | 8,4 | 8,2 | 0,2 | 0,04 |
| Solyc05g046020.1.1 | Peroxidase | 6,9 | 7,7 | -0,8 | 0,01 |
| Solyc05g046290.1.1 | Xyloglucan endotransglucosylase/hydrolase 2 | 7,1 | 7,0 | 0,1 | 0,14 |
| Solyc07g006560.1.1 | Hypersensitive response assisting protein | 6,0 | 7,0 | -1,0 | 0,02 |
| Solyc05g050130.1.1 | Acidic chitinase | 10,7 | 9,9 | 0,9 | 0,00 |
| Solyc05g050880.1.1 | Peroxidase | 9,0 | 9,2 | -0,2 | 0,05 |
| Solyc07g044840.1.1 | 2 3-bisphosphoglycerate-independent phosphoglycerate mutase | 8,7 | 7,2 | 1,4 | 0,00 |
| Solyc05g052280.1.1 | Peroxidase | 8,2 | 8,9 | -0,7 | 0,00 |
| Solyc05g052400.1.1 | Laccase | 6,0 | 6,7 | -0,7 | 0,04 |
| Solyc07g062210.1.1 | Os06g0207500 protein | 8,7 | 9,5 | -0,7 | 0,00 |
| Solyc08g074620.1.1 | Polyphenol oxidase | 6,5 | 7,5 | -1,1 | 0,01 |
| Solyc06g005940.1.1 | Protein disulfide isomerase | 7,4 | 6,0 | 1,4 | 0,00 |
| Solyc08g075390.1.1 | Isopentenyl-diphosphate delta-isomerase family protein | 7,7 | 7,4 | 0,3 | 0,06 |
| Solyc06g007340.1.1 | Gamma-interferon-inducible lysosomal thiol reductase | 7,1 | 8,2 | -1,1 | 0,01 |
| Solyc09g010930.1.1 | NAD-dependent epimerase/dehydratase | 6,6 | 7,2 | -0,6 | 0,06 |
| Solyc06g009110.1.1 | Nucellin-like aspartic protease | 7,0 | 7,6 | -0,6 | 0,01 |
| Solyc11g011960.1.1 | UTP-glucose 1 phosphate uridylyltransferase | 6,0 | 7,1 | -1,0 | 0,01 |
| Solyc06g050130.1.1 | Alpha-galactosidase-like protein | 6,0 | 7,5 | -1,5 | 0,00 |
| Solyc06g050440.1.1 | Peroxidase | 8,2 | 9,4 | -1,2 | 0,00 |
| Solyc06g053380.1.1 | Chitinase | 6,0 | 6,9 | -0,8 | 0,01 |
| Solyc06g060970.1.1 | Expansin-like protein | 6,0 | 7,4 | -1,3 | 0,00 |
| Solyc06g062280.1.1 | DSBA oxidoreductase | 6,3 | 7,6 | -1,2 | 0,00 |
| Solyc06g062380.1.1 | Acid phosphatase | 6,3 | 8,1 | -1,8 | 0,00 |
| Solyc06g065530.1.1 | GDSL esterase/lipase At1g29670 | 6,0 | 7,8 | -1,7 | 0,00 |
| Solyc06g065720.1.1 | DOMON domain-containing protein | 6,0 | 6,5 | -0,5 | 0,02 |
| Solyc06g068860.1.1 | Alpha-mannosidase | 9,5 | 9,0 | 0,5 | 0,03 |
| Solyc12g014440.1.1 | BNR/Asp-box repeat protein | 6,8 | 6,3 | 0,4 | 0,06 |
| Solyc06g072220.1.1 | Kunitz trypsin inhibitor | 8,7 | 9,8 | -1,1 | 0,00 |
| Solyc06g072230.1.1 | Kunitz trypsin inhibitor | 8,8 | 9,6 | -0,8 | 0,00 |
| Solyc12g056960.1.1 | Glucan 1 3-beta-glucosidase | 9,9 | 10,0 | 0,0 | 0,13 |
| Solyc00g006800.1.1 | Transaldolase | 8,7 | 8,2 | 0,5 | 0,00 |
| Solyc06g073760.1.1 | Beta-D-glucosidase | 7,6 | 7,4 | 0,1 | 0,06 |
| Solyc06g074850.1.1 | Serine carboxypeptidase | 7,9 | 8,9 | -1,0 | 0,00 |
| Solyc01g097460.1.1 | Ribose-5-phosphate isomerase | 8,2 | 7,9 | 0,3 | 0,04 |
| Solyc06g082240.1.1 | Laccase-13 | 6,0 | 8,2 | -2,2 | 0,00 |
| Solyc06g082420.1.1 | Peroxidase 3 | 8,9 | 8,8 | 0,2 | 0,07 |
| Solyc06g083030.1.1 | Serine carboxypeptidase 1 | 8,3 | 8,6 | -0,2 | 0,01 |
| Solyc06g083040.1.1 | Serine carboxypeptidase 1 | 8,6 | 9,0 | -0,5 | 0,00 |
| Solyc01g111120.1.1 | Triosephosphate isomerase | 8,2 | 6,0 | 2,2 | 0,00 |
| Solyc07g005090.1.1 | Chitinase A | 8,2 | 8,7 | -0,5 | 0,01 |
| Solyc07g005100.1.1 | Chitinase-like protein | 9,8 | 8,7 | 1,2 | 0,00 |
| Solyc07g005330.1.1 | Glucan endo-1 3-beta-glucosidase | 6,6 | 8,3 | -1,7 | 0,00 |
| Solyc07g005960.1.1 | Serine carboxypeptidase K10B2.2 | 6,5 | 8,2 | -1,7 | 0,00 |
| Solyc02g024050.1.1 | Ferredoxin--NADP reductase | 8,4 | 8,2 | 0,2 | 0,06 |
| Solyc07g006570.1.1 | S8-RNase | 8,8 | 9,1 | -0,3 | 0,01 |
| Solyc07g006860.1.1 | Xyloglucan endotransglucosylase/hydrolase 3 | 8,1 | 9,5 | -1,4 | 0,00 |
| Solyc07g006870.1.1 | Xyloglucan endotransglucosylase/hydrolase 8 | 8,3 | 7,7 | 0,6 | 0,00 |
| Solyc07g007550.1.1 | Heparanase | 8,7 | 9,3 | -0,6 | 0,00 |
| Solyc07g007670.1.1 | Purple acid phosphatase 3 | 8,0 | 8,3 | -0,3 | 0,01 |
| Solyc02g083810.1.1 | Ferredoxin--NADP reductase | 7,1 | 6,4 | 0,7 | 0,00 |
| Solyc07g041900.1.1 | Cathepsin L-like cysteine proteinase | 9,2 | 8,8 | 0,3 | 0,01 |
| Solyc07g043240.1.1 | Pectinesterase | 6,5 | 8,1 | -1,6 | 0,00 |
| Solyc04g011510.1.1 | Triosephosphate isomerase | 7,5 | 7,4 | 0,1 | 0,14 |
| Solyc07g045440.1.1 | Fasciclin-like arabinogalactan protein 19 | 8,8 | 9,1 | -0,3 | 0,00 |
| Solyc07g047740.1.1 | Peroxidase | 7,6 | 8,5 | -0,9 | 0,00 |
| Solyc05g008600.1.1 | Fructose-bisphosphate aldolase | 7,1 | 6,8 | 0,3 | 0,09 |
| Solyc05g052600.1.1 | Fructose-1 6-bisphosphatase class 1 | 7,7 | 6,9 | 0,8 | 0,02 |
| Solyc06g083190.1.1 | Peptidyl-prolyl cis-trans isomerase | 6,0 | 6,5 | -0,5 | 0,01 |
| Solyc07g052510.1.1 | Peroxidase | 9,6 | 9,8 | -0,3 | 0,01 |
| Solyc07g053540.1.1 | Fasciclin-like arabinogalactan protein 4 | 7,5 | 7,8 | -0,4 | 0,06 |
| Solyc07g063740.1.1 | Ferredoxin-like | 7,8 | 7,9 | -0,1 | 0,09 |
| Solyc09g009260.1.1 | Fructose-bisphosphate aldolase | 6,5 | 8,0 | -1,5 | 0,00 |
| Solyc11g069040.1.1 | Lactoylglutathione lyase | 8,6 | 7,5 | 1,1 | 0,00 |
| Solyc07g065090.1.1 | Polygalacturonase inhibitor protein | 6,0 | 6,7 | -0,7 | 0,03 |
| Solyc07g065110.1.1 | Protease inhibitor/seed storage/lipid transfer protein family protein | 8,1 | 7,7 | 0,3 | 0,01 |
| Solyc07g065120.1.1 | Glycerophosphoryl diester phosphodiesterase family protein | 9,2 | 8,7 | 0,5 | 0,01 |
| Solyc08g005000.1.1 | Glucan endo-1 3-beta-glucosidase 3 | 8,7 | 8,7 | 0,0 | 0,13 |
| Solyc08g006790.1.1 | Early nodulin-55-1 | 7,3 | 8,1 | -0,7 | 0,01 |
| Solyc08g006850.1.1 | Patatin-like phospholipase family protein | 6,7 | 6,0 | 0,7 | 0,05 |
| Solyc08g007670.1.1 | Subtilisin-like protease | 8,9 | 9,0 | -0,1 | 0,11 |
| Solyc08g014380.1.1 | Pectinacetylesterase | 6,9 | 7,9 | -1,0 | 0,01 |
| Solyc02g069630.1.1 | Subtilisin-like serine protease | 6,8 | 7,6 | -0,8 | 0,01 |
| Solyc08g061060.1.1 | Blight-associated protein P12 | 6,0 | 7,8 | -1,7 | 0,00 |
| Solyc02g077040.1.1 | Cathepsin B-like cysteine proteinase 5 | 9,2 | 8,2 | 1,0 | 0,00 |
| Solyc08g066810.1.1 | Glycosyl hydrolase family 5 protein/cellulase | 7,9 | 8,6 | -0,7 | 0,00 |
| Solyc03g119690.1.1 | Aspartic proteinase nepenthesin-1 | 7,3 | 8,1 | -0,8 | 0,01 |
| Solyc08g067500.1.1 | Non-specific lipid-transfer protein | 7,3 | 8,0 | -0,7 | 0,03 |
| Solyc08g068420.1.1 | FAD-linked oxidoreductase | 6,4 | 7,6 | -1,1 | 0,00 |
| Solyc03g123900.1.1 | Mannosyl-oligosaccharide 1 2-alpha-mannosidase | 7,3 | 8,1 | -0,7 | 0,01 |
| Solyc04g082710.1.1 | Cathepsin B-like cysteine proteinase 3 | 8,3 | 9,5 | -1,2 | 0,00 |
| Solyc05g054710.1.1 | Beta-hexosaminidase b | 8,4 | 8,0 | 0,5 | 0,01 |
| Solyc08g079090.1.1 | Laccase-22 | 8,2 | 8,5 | -0,3 | 0,02 |
| Solyc08g079860.1.1 | Subtilisin-like protease | 9,1 | 7,5 | 1,6 | 0,00 |
| Solyc08g079870.1.1 | Subtilisin-like protease | 10,8 | 8,5 | 2,3 | 0,00 |
| Solyc08g079900.1.1 | Subtilisin-like protease | 10,1 | 8,0 | 2,1 | 0,00 |
| Solyc08g080590.1.1 | Osmotin 81 | 9,7 | 9,0 | 0,7 | 0,00 |
| Solyc08g080620.1.1 | Osmotin-like protein | 9,7 | 9,3 | 0,4 | 0,01 |
| Solyc08g080640.1.1 | Osmotin-like protein | 8,3 | 8,7 | -0,4 | 0,02 |
| Solyc08g080650.1.1 | Osmotin-like protein | 8,9 | 8,3 | 0,6 | 0,01 |
| Solyc07g064880.1.1 | Small ubiquitin-related modifier | 6,4 | 7,7 | -1,3 | 0,00 |
| Solyc08g082860.1.1 | Alpha alpha-trehalase | 7,5 | 8,5 | -0,9 | 0,00 |
| Solyc08g083210.1.1 | Endoglucanase 1 | 7,0 | 7,1 | -0,1 | 0,14 |
| Solyc09g006010.1.1 | Pathogenesis related protein PR-1 | 9,1 | 8,9 | 0,2 | 0,09 |
| Solyc09g007010.1.1 | Pathogenesis related protein PR-1 | 9,6 | 6,3 | 3,3 | 0,00 |
| Solyc09g007020.1.1 | Pathogenesis-related protein | 8,7 | 6,0 | 2,7 | 0,00 |
| Solyc09g007520.1.1 | Peroxidase | 9,2 | 9,4 | -0,2 | 0,04 |
| Solyc09g007660.1.1 | Fasciclin-like arabinogalactan protein 7 | 7,6 | 8,5 | -0,9 | 0,01 |
| Solyc08g067100.1.1 | Aspartic proteinase nepenthesin-1 | 9,7 | 10,2 | -0,5 | 0,00 |
| Solyc11g005670.1.1 | Ubiquitin | 7,4 | 7,6 | -0,1 | 0,11 |
| Solyc11g008810.1.1 | Beta-hexosaminidase b | 6,0 | 6,8 | -0,8 | 0,01 |
| Solyc12g010040.1.1 | Leucyl aminopeptidase | 8,4 | 7,6 | 0,8 | 0,00 |
| Solyc09g014480.1.1 | Polygalacturonase inhibitor protein | 6,6 | 7,2 | -0,6 | 0,00 |
| Solyc01g015080.1.1 | Peroxidase | 10,2 | 9,9 | 0,3 | 0,06 |
| Solyc09g015840.1.1 | Receptor-like kinase | 6,0 | 7,9 | -1,8 | 0,00 |
| Solyc09g072660.1.1 | FAD-binding domain-containing protein | 9,4 | 9,4 | 0,0 | 0,13 |
| Solyc09g072700.1.1 | Peroxidase 57 | 7,8 | 7,0 | 0,8 | 0,02 |
| Solyc01g105070.1.1 | Peroxidase | 8,1 | 8,6 | -0,5 | 0,00 |
| Solyc09g075730.1.1 | Receptor-like kinase | 6,0 | 7,3 | -1,3 | 0,00 |
| Solyc09g075810.1.1 | Blue copper protein | 9,1 | 9,4 | -0,3 | 0,00 |
| Solyc09g090020.1.1 | Germin-like protein 5 | 6,0 | 6,9 | -0,9 | 0,01 |
| Solyc05g050890.1.1 | Peroxidase | 9,2 | 8,9 | 0,3 | 0,03 |
| Solyc06g076630.1.1 | Peroxidase | 9,5 | 9,7 | -0,2 | 0,01 |
| Solyc02g088390.1.1 | Blue copper-like protein | 6,0 | 8,3 | -2,3 | 0,00 |
| Solyc05g054760.1.1 | Dehydroascorbate reductase | 8,9 | 8,8 | 0,0 | 0,14 |
| Solyc06g009020.1.1 | Glutathione S-transferase | 9,7 | 8,3 | 1,4 | 0,00 |
| Solyc06g048410.1.1 | Superoxide dismutase | 8,8 | 8,5 | 0,3 | 0,03 |
| Solyc09g098340.1.1 | Aspartic proteinase-like protein 2 | 8,3 | 8,2 | 0,1 | 0,04 |
| Solyc09g098540.1.1 | Chitinase-like protein | 7,5 | 6,4 | 1,1 | 0,01 |
| Solyc10g005960.1.1 | Fasciclin-like arabinogalactan protein 10 | 9,2 | 9,5 | -0,3 | 0,00 |
| Solyc10g006290.1.1 | Endoribonuclease L-PSP family protein | 7,7 | 8,0 | -0,3 | 0,09 |
| Solyc10g007070.1.1 | CT099 | 7,9 | 8,2 | -0,3 | 0,01 |
| Solyc10g009410.1.1 | Aspartic proteinase nepenthesin I | 6,0 | 7,3 | -1,2 | 0,00 |
| Solyc10g038130.1.1 | Pectinacetylesterase like protein | 8,6 | 9,1 | -0,5 | 0,00 |
| Solyc10g052880.1.1 | Leucine-rich repeat family protein | 9,2 | 10,1 | -0,9 | 0,00 |
| Solyc10g055800.1.1 | Chitinase | 9,6 | 9,4 | 0,1 | 0,05 |
| Solyc10g055810.1.1 | Endochitinase | 9,6 | 8,1 | 1,5 | 0,00 |
| Solyc10g055820.1.1 | Chitinase | 8,5 | 6,6 | 1,8 | 0,00 |
| Solyc10g074440.1.1 | Endochitinase | 8,7 | 9,0 | -0,2 | 0,05 |
| Solyc10g074820.1.1 | Unknown Protein | 6,4 | 7,2 | -0,8 | 0,04 |
| Solyc10g074920.1.1 | Mannan endo-1 4-beta-mannosidase | 8,2 | 9,2 | -1,0 | 0,00 |
| Solyc10g075100.1.1 | Non-specific lipid-transfer protein | 8,1 | 8,2 | -0,1 | 0,08 |
| Solyc10g075110.1.1 | Non-specific lipid-transfer protein | 8,1 | 8,8 | -0,7 | 0,02 |
| Solyc10g076220.1.1 | Peroxidase 1 | 8,1 | 8,2 | -0,1 | 0,09 |
| Solyc10g079860.1.1 | Beta-1 3-glucanase | 10,3 | 9,5 | 0,7 | 0,00 |
| Solyc10g081120.1.1 | Alpha-L-arabinofuranosidase | 9,7 | 9,7 | 0,1 | 0,09 |
| Solyc10g081190.1.1 | Leucine-rich repeat receptor-like kinase | 7,3 | 8,0 | -0,6 | 0,04 |
| Solyc08g066740.1.1 | CT099 | 7,8 | 7,9 | -0,1 | 0,04 |
| Solyc09g009390.1.1 | Monodehydroascorbate reductase | 8,0 | 7,1 | 0,8 | 0,01 |
| Solyc10g084240.1.1 | Peroxidase | 8,7 | 8,2 | 0,5 | 0,00 |
| Solyc10g084320.1.1 | Subtilisin-like protease | 6,3 | 7,4 | -1,1 | 0,00 |
| Solyc09g091840.1.1 | Glutathione-disulfide reductase | 7,4 | 6,0 | 1,4 | 0,00 |
| Solyc10g084400.1.1 | Glutathione S-transferase | 8,8 | 7,3 | 1,5 | 0,00 |
| Solyc10g085670.1.1 | LRR receptor-like serine/threonine-protein kinase FEI 1 | 7,0 | 7,8 | -0,8 | 0,02 |
| Solyc11g005480.1.1 | Citrate binding protein | 7,2 | 6,0 | 1,2 | 0,01 |
| Solyc12g008640.1.1 | Gamma-glutamyltransferase-like protein | 6,0 | 7,4 | -1,4 | 0,01 |
| Solyc02g063070.1.1 | 14-3-3 protein beta/alpha-1 | 6,4 | 7,7 | -1,3 | 0,00 |
| Solyc11g010120.1.1 | Peroxidase 17 | 8,6 | 8,6 | 0,0 | 0,15 |
| Solyc11g011020.1.1 | Receptor-like protein kinase 1-like | 6,4 | 8,1 | -1,7 | 0,00 |
| Solyc11g012580.1.1 | Glucan endo-1 3-beta-glucosidase 3 | 6,0 | 7,3 | -1,3 | 0,00 |
| Solyc11g012870.1.1 | LysM domain containing protein | 8,7 | 8,7 | -0,1 | 0,09 |
| Solyc09g015830.1.1 | Receptor protein kinase-like protein | 7,3 | 7,8 | -0,5 | 0,09 |
| Solyc11g018800.1.1 | Peroxidase 2 | 6,4 | 8,3 | -1,9 | 0,00 |
| Solyc11g040330.1.1 | Endo-1 4-beta-xylanase | 7,7 | 8,5 | -0,8 | 0,00 |
| Solyc11g066250.1.1 | Serine carboxypeptidase | 8,3 | 8,7 | -0,4 | 0,00 |
| Solyc11g066290.1.1 | Icc family phosphohydrolase | 8,1 | 9,1 | -1,0 | 0,00 |
| Solyc00g098560.1.1 | GDSL esterase/lipase At5g03980 | 6,0 | 7,1 | -1,0 | 0,00 |
| Solyc11g066520.1.1 | Serine carboxypeptidase family protein expressed | 7,6 | 8,5 | -0,9 | 0,00 |
| Solyc11g066620.1.1 | Aspartyl protease family protein | 7,9 | 7,9 | -0,1 | 0,13 |
| Solyc11g068440.1.1 | Glucan endo-1 3-beta-glucosidase 7 | 6,0 | 7,4 | -1,4 | 0,00 |
| Solyc01g098650.1.1 | GDSL esterase/lipase At1g54790 | 8,8 | 9,7 | -0,8 | 0,00 |
| Solyc02g086700.1.1 | Beta-1 3-glucanase | 10,8 | 9,4 | 1,5 | 0,00 |
| Solyc12g005720.1.1 | Cysteine-rich receptor-like protein kinase | 10,3 | 10,1 | 0,2 | 0,03 |
| Solyc12g008580.1.1 | Glucan endo-1 3-beta-glucosidase 4 | 8,3 | 8,6 | -0,3 | 0,02 |
| Solyc04g015190.1.1 | Glucan endo-1 3-beta-glucosidase 5 | 7,5 | 8,1 | -0,6 | 0,03 |
| Solyc12g009800.1.1 | Purple acid phosphatase 3 | 6,0 | 7,9 | -1,8 | 0,00 |
| Solyc08g074390.1.1 | Glucan endo-1 3-beta-glucosidase 6 | 8,1 | 9,1 | -1,0 | 0,00 |
| Solyc12g013900.1.1 | CT099 | 8,7 | 8,6 | 0,1 | 0,09 |
| Solyc12g014270.1.1 | Peptide-N4- | 7,5 | 7,7 | -0,2 | 0,08 |
| Solyc12g014420.1.1 | Glucan endo-1 3-beta-glucosidase A6 | 9,5 | 9,8 | -0,3 | 0,00 |
| Solyc09g090970.1.1 | Major allergen Mal d 1 | 9,0 | 6,0 | 3,0 | 0,00 |
| Solyc12g017460.1.1 | GDSL esterase/lipase At1g28590 | 7,5 | 8,9 | -1,4 | 0,00 |
| Solyc12g019890.1.1 | Glucan endo-1 3-beta-glucosidase 5 | 7,5 | 7,9 | -0,4 | 0,00 |
| Solyc12g042380.1.1 | MtN19-like protein | 8,1 | 6,0 | 2,1 | 0,00 |
| Solyc12g044450.1.1 | Os06g0207500 protein | 6,0 | 7,4 | -1,4 | 0,00 |
| Solyc09g090980.1.1 | Major allergen Mal d 1 | 9,7 | 7,3 | 2,4 | 0,00 |
| Solyc12g056390.1.1 | Thaumatin-like protein | 6,0 | 6,0 | 0,0 | 0,14 |
| Solyc12g088670.1.1 | Cathepsin B-like cysteine proteinase | 8,6 | 8,3 | 0,3 | 0,00 |
| Solyc12g088760.1.1 | Subtilisin-like protease | 9,7 | 9,6 | 0,1 | 0,12 |
| Solyc12g095910.1.1 | Vacuolar processing enzyme-3 | 6,0 | 7,6 | -1,6 | 0,00 |
| Solyc12g098540.1.1 | Ectonucleoside triphosphate diphosphohydrolase 6 | 7,3 | 6,7 | 0,7 | 0,05 |
| Solyc12g099160.1.1 | Serine carboxypeptidase K10B2.2 | 8,5 | 8,7 | -0,2 | 0,04 |
| Solyc12g100120.1.1 | Acetyl-CoA carboxylase-like protein | 9,3 | 9,1 | 0,2 | 0,09 |
